# Loss of miR-122 promotes cell migration and poor prognosis in triple-negative breast cancer treated by neoadjuvant chemotherapy

**DOI:** 10.1007/s12094-025-04082-x

**Published:** 2025-10-22

**Authors:** Mauricio Flores-Fortis, Isidro X. Perez-Añorve, Carlos C. Patiño-Morales, Ernesto Soto-Reyes, Claudia H. Gonzalez-De la Rosa, Joaquin Manzo-Merino, Arturo Aguilar-Rojas, Nicolas Villegas, Elena Arechaga-Ocampo

**Affiliations:** 1 Posgrado en Ciencias Naturales e Ingenieria, Unidad Cuajimalpa, Universidad Autonoma Metropolitana, 05348, Mexico City, Mexico; 2 Departamento de Ciencias Naturales, Unidad Cuajimalpa, Universidad Autonoma Metropolitana, 05348 Mexico City, Mexico; 3 Laboratorio de Biología del Desarrollo y Teratogenesis Experimental, Hospital Infantil de Mexico Federico Gomez, 06720 Mexico City, Mexico; 4 Catedras CONAHCyT-Division de Investigacion Basica, Instituto Nacional de Cancerologia, 14080 Mexico City, Mexico; 5 Unidad de Investigacion Medica en Medicina Reproductiva, Unidad Médica de Alta Especialidad No. 4 Luis Castelazo-Ayala, IMSS, 01090 Mexico City, Mexico; 6 Departamento de Biomedicina Molecular, Centro de Investigacion y de Estudios Avanzados, 07360 Mexico City, Mexico; 7 Red de Investigación en Salud, Universidad Autonoma Metropolitana, Mexico City, Mexico

**Keywords:** Tumor suppressor, miR-122, BORIS, Chemotherapy, TNBC

## Abstract

**Background:**

MicroRNAs (miRNAs) are short non-coding RNAs that regulate gene expression post-transcriptionally. miR-122 is abnormally expressed in breast cancer, functioning as both a tumor suppressor and oncomiR, however, its role in triple-negative breast cancer (TNBC) remains unclear.

**Purpose:**

In this study we investigate the molecular mechanism of miR-122 in TNBC patients undergoing neoadjuvant chemotherapy (NAC).

**Methods:**

We analyzed the expression of miR-122 and its clinical association in TNBC patients from TCGA and KM-plotter datasets. Functional analysis was performed by modulating miR-122 levels in TNBC cells through antagomiR transfections for knockdown assays, and mimic-miR-122 assays, followed by RT-qPCR, immunoblotting, cell viability and migration assays.

**Results:**

Downregulation of miR-122 in TNBC patients is associated with tumor recurrence but not with pathologic complete response after NAC. Low miR-122 levels in rapid-relapse TNBC patients activate a gene co-expression network enriched in genes linked to migration, invasion, and cell differentiation. Among these, DNA-binding protein BORIS was identified as a target of miR-122. Overexpression of miR-122 inhibited cell migration and BORIS expression. Additionally, BORIS promoted a gene expression profile related to cell differentiation and cytoskeletal components in TNBC tumors deficient of miR-122.

**Conclusion:**

The loss of miR-122 in TNBC tumors is associated with rapid relapse after chemotherapy in patients and allows cell migration by promoting a metastatic-related transcriptomic landscape, including positive modulation of BORIS expression.

**Supplementary Information:**

The online version contains supplementary material available at 10.1007/s12094-025-04082-x.

## Introduction

MicroRNA-122 (miR-122) is aberrantly expressed in several malignancies, in which it has been proposed as diagnostic or prognostic biomarker for human cancer [1–6] and it has been linked to enhances patient response to standard therapy [7–9]. miR-122 has a role in chemotherapy response of the hepatocellular carcinoma (HCC) [10, 11], Lymph Node Carcinoma of the Prostate (LNCap) [12] and breast cancer [13, 14]. The molecular function of mir-122 is related to the regulation of genes associated with proliferation, apoptosis [10, 15–17], tumor metabolism [11, 18], drug metabolic enzymes and transporters [19], and the epithelial-mesenchymal transition (EMT) [20]. In breast cancer, miR-122 has been reported as tumor suppressor [16] and oncomiR [21], however, in Triple Negative Breast Cancer (TNBC), there is still no related evidence explaining the role of miR-122 and its association with chemotherapy treatment.

Loss of tumor suppressor miRNAs expression results in increased expression of genes for tumor progression, including antiapoptotic proteins or transcription factors [22, 23]. The Brother Of the Regulator of Imprinted Sites (BORIS) protein, encoded by the *CTCFL* gene, is a DNA-binding protein involved in chromatin insulation, genomic imprinting, intra/interchromosomal interactions, and global three-dimensional genome organization. [24–29]. Mainly, BORIS is mainly expressed in the germline, and is aberrantly activated in many tumors reporting as cancer germline gene or cancer testis antigen [29]. There is now accumulating evidence pointing to a role for BORIS in the progression, or metastatic potential of cancers via its ability to transcriptionally reprogram cells [30]. Moreover, previous studies suggest a role of BORIS in drug resistance through the maintenance of a stemness state [31] and the regulation of epigenetic processes [32]. However, currently no miRNA capable of regulating BORIS expression has been reported, nor has it been determined whether BORIS expression is dysregulated in drug response of TNBC.

In this work, we aimed to determine the role of miR-122 in TNBC patients treated with NAC. We observed that TNBC patients with loss of miR-122 expression are prone to early relapse. We identify differentially expressed genes (DEGs) and gene co-expression network related to differentiation, stemness, and migration pathways in TNBC patients with loss of miR-122 expression and worse outcome. *BORIS* gene was identified as target of miR-122. Mechanistically, differential expression of miR-122 regulated cell migration and modulated the expression of BORIS. These results showed that loss of miR-122 expression promotes a gene expression profile associated to cellular invasion and migration relative to worse prognosis in TNBC patients submitted to NAC. Loss of miR-122 expression might favor cell migration at least in part by modulates expression of BORIS.

## Material and methods

### TNBC patient population and TNBC datasets and data preprocessing

Clinical data and miRNAs and mRNA expression datasets from TNBC patient were downloaded from TCGA-BRCA project (Illumina HiSeq platform) from The Cancer Genome Atlas (TCGA) database (https://portal.gdc.cancer.gov). We included 105 TNBC patients with I-III clinical stage without distant metastasis. Cases with incomplete clinical information, missing any miRNA and mRNA data, and cases that received radiation or endocrine therapy were excluded.

The gene expression profiles of the tumor samples contained 1846 miRNAs genes and 54,611 mRNA transcripts. Total TNBC population was stratified according to higher or lower than median expression levels of miR-122 or *BORIS* gene. Recurrence-free survival (RFS) of patients was determined using the datasets from Kaplan-Meier plotter (www.kmplot.com) and the differences between the curves were analyzed by two-sided log rank tests. The expression of miR-122 and *BORIS* gene was dichotomized to pathologic complete response (pCR) and pathological partial response/pathological non-response (pPR/pNR). A P value < 0.05 was considered statistically significant.

### Differential gene expression analysis

DEGs in TNBC tumor samples were categorized as follow: a) miR-122 downregulated relative to samples with miR-122 upregulated; or b) *BORIS* gene overexpression with respect to samples with *BORIS* downregulated. The statistical analysis were performed using the Bioconductor R package “DESeq2” version 1.38.3 [33]. The lowly expressed genes (read counts <10 in at least 2 samples) were removed using the DESeq2 package. To remove composition biases between the libraries the median of ratios method was used for normalization. The statistically significant DEGs were filtered based on the cut-off criteria as false discovery rate threshold (FDR) < 0.05 and |log2 FC| > 1.5.

### Co-expression network construction and module eigengenes detection

To build the co-expression network, DEGs were utilized. Weighted gene co-expression network analysis (WGCNA) was performed by the Bioconductor R package “WGCNA” version 1.70–3.70 [34]. The sample outliers were removed by hierarchical clustering analysis using the flashClust tool included in the R package WGCNA. Then, a scale-free co-expression networks of all genes were constructed using the following parameters: power (β) = 9, minModuleSize = 30, deepSplit = 0, neworkType = "signed”. Then, genes were hierarchically clustered using 1-TOM as the distance measure and modules were determined by choosing a height cutoff 0.80 for the resulting dendrogram. Highly similar modules were identified by clustering and merged. The feature gene network was then shown after we computed module eigengene (ME), the gene significance (GS) and module membership (MM). Finally, the correlations between ME values and down- and up-regulation of miR-122 and BORIS gene over- and downregulated were calculated. The statistically significant modules were identified by P<0.05.

### Construction of protein–protein interaction (PPI) network and detection of hub genes

The interaction pattern of genes from the blue module (*P* < 0.05) was predicted using the Search Tool for the Retrieval of Interacting Genes/Proteins (STRING) database (https://string-db.org) with a confidence cutoff of > 0.7. The protein-protein interaction (PPI) network was constructed and visualized by Cytoscape software (version 3.8.2) [35]. The hub genes from blue module were identified by the Molecular Complex Detection (MCODE) [36] score using Cytoscape version 3.9.1. The hub genes were selected with a cutoff score > 10. Color and shape of nodes were adjusted based on MCODE score.

### Functional enrichment analysis

Functional enrichment analyzes were performed by Gene Ontology (GO), Kyoto Encyclopedia of Genes and Genomes (KEGG) pathway, Reactome pathways and WikiPathways enrichment analysis with the “STRING enrichment” app using Cytoscape version 3.9.1 [37]. Only the top 10 terms with a *P* < 0.05 were extracted. The R package “GOplot” [38] version 1.0.2 was used to visualize and plot the Significance (−1*log10(adjust P value)) and log2 Fold Change enrichment (log2FCe) of each of terms.

### Identification of miR-122 target genes

The miR-122 target genes were predicted by miR-TarBase (http://mirtarbase.mbc.nctu.edu.tw), miRDB (http://mirdb.org), TargetScan Human 7.2 (https://www.targetscan.org/vert_80/) and miRmap (https://mirmap.ezlab.org) prediction tools. The miR-target interactions identified by at least three algorithms were considered.

### Cell culture

Human TNBC cell line MDA-MB-231 was obtained from ATCC (#HTB-26). Radioresistant MDA-MB-231 cell line (MDA-MB-231RR) was established previously in our laboratory as describe Perez-Añorve et al. [21]. The MCF-10A cell line was employed as non-tumorigenic human breast cells. MDA-MB-231 and MDA-MB-231RR cells were cultured in Dulbecco Modified Eagle Medium (DMEM) (Gibco, Thermo Fisher Scientific Inc, MA, USA); while MCF-10A was maintained in DMEM/F12 (Invitrogen, CA, USA) cell media. Cultures were supplemented with 10% fetal bovine serum (FBS) and penicillin and streptomycin at 37 °C in a 5% CO_2_ atmosphere.

### Drug treatment and cell viability assay

Doxorubicin was purchased from Pfizer Inc (NY, USA). Cyclophosphamide (ALX-400-051-G005), docetaxel (BML-T129-0005), and cisplatin (ALX-400-040-M050) were purchased from Enzo Life Science, Inc (NY, USA). All drugs were prepared at stock dilution of 1mg/mL and were diluted to the required concentration with DMEM when needed. The half-maximal inhibitory concentration (IC_50_) of each drug was determinate to 24 h. Cytotoxic effect of each drug on MDA-MB-231 and MDA-MB-231RR cells was determined by MTT [3-(4,5-dimethylthiazol-2-yl)−2,5-diphenyltetrazolium bromide (≥97% purity; Sigma-Aldrich; Merck KGaA)] assay. The absorbance was then measured at 450 nm using Multiskan^TM^ FC plate reader (Thermo Fisher Scientific Inc, MA, USA). Each experimental condition was performed in triplicate and repeated at least twice. All values were normalized with respect to the viability of untreated cells. miR-122 expression also was evaluated by RT-qPCR after drug treatment.

### RT-qPCR

The expression of miR-122 and *BORIS* gene was evaluated via two-step RT-qPCR. The TaqMan® MicroRNA Assay (Applied Biosystems, MA, USA) was used for miR-122 analysis. Total RNA (100 ng) was retro-transcribed using the hsa-miR-122-5p primer (Assay ID: 002245) according to the manufacturer’s instructions, and detection of miR-122 was performed using TaqMan Universal PCR Master Mix (Applied Biosystems, MA, USA). For *BORIS*, 200 ng of total RNA were retro-transcribed using Go Taq® Probe system (Promega Corp., WI, USA), and qPCR was performing using Maxima SYBR Green/Rox qPCR (Thermo Fisher Scientific Inc, MA, USA) and primers: Fwd 5´-GGAGCATTTGTAAACAGTCGGG-3´; Rvs 5´-ATGACCGCTCACATTCGTACC-3´. RNU44 (Assay ID: 001094) and *GAPDH* (Fwd 5´-TGCACCACCAACTGCTTAGC-3; Rvs 5´-GGCTGGACTGTGGTCATGAG-3´) were used as the reference genes for miR-122 and *BORIS* genes, respectively. qPCR reaction was carried out using an ABI Applied Biosystems 7500 Real-Time PCR System (Applied Biosystems, MA, USA). The relative expression level of miR-122 and *BORIS* was calculated using 2-ΔΔCt method.

### Transfection assay

MDA-MB-231 and MDA-MB-231RR cells were seeded at a density of 80 x 10^3^ cells/well into 12-well plates. After 24 h MDA-MB-231 and MDA-MB-231RR cells were transfected with 10 nM hsa-miR-122-5p mimics (#4464066, Ambion, MA, USA) and 30 nM hsa-miR-122-5p inhibitor (#4464084, Ambion, MA, USA), respectively, using Lipofectamine 2000 (Invitrogen, CA, USA) according to the manufacturer’s instructions and diluted in Opti-Mem® reduced serum media (Gibco, MA, USA). Scramble sequence (#AM170010, Ambion, MA, USA) was used as negative control of transfection. After 24 h of transfection total RNA and proteins were isolated. Transfection efficiency was evaluated by expression levels of miR-122 by RT-qPCR as previously described [21].

### Wound-healing assay

MDA-MB-231 cells were seeded in 6-well dishes at a density of 2 × 10^5^ cells/well. After 24 h, MDA-MB-231 cells were transfected with hsa-miR-122-5p mimics. After 24 h of transfection, a scratch in the cell monolayer was made using micropipette tip. After wounding, images were captured using the inverted microscope Axiovert 40 CFL (Zeiss, BW, Germany) at 0, 6, 24, 30, and 48 h. The gap size was measured with the ImageJ software version 1.53 (National Institutes of Health, MD, USA) and the percentage migration was calculated from the following equation [39]: Wound closure (%) = (*Wt*_*0*_ - *Wt*_*i*_) ÷ *Wt*_*0*_ × 100, where *Wt*_*0*_ is the initial wound area at the outset of the experiment, and *Wt*_*i*_ is the measured wound area at a given time interval. Each experimental condition was performed in duplicate and repeated at least twice. Scramble sequence was used as negative control of transfection.

### Transwell cell migration assay

A migration experiment was conducted in 24-well transwells with a pore size of 8.0 μM (Corning Inc., USA). Briefly, 1×10^5^ cells/chamber of MDA-MB-231 cells, transfected or not with 10 nM hsa-miR-122-5p mimics, suspended in 200 μl FBS-free medium were transferred to the upper chambers separately. In each experimental condition, 600 μl of a medium containing 10% FBS as a chemoattractant were added to the bottom compartment. Non-migrated cells at the top of the transwells were manually removed using a cotton-tipped applicator after 48 h or 72 h of incubation at 37 °C and 5% CO_2_. In contrast, migrated cells on the lower surface were fixed and stained with 0.2% crystal violet solution to take microphotographs, or lysed with the CytoTox 96® non-radioactive cytotoxicity assay (Promega, USA) to detect the migrated cells based on LDH activity according to the manufacturer’s instructions. The absorbance was then measured at 492 nm using Multiskan^TM^ FC plate reader (Thermo Fisher Scientific Inc, MA, USA). Each experimental condition was performed in triplicate and repeated at least twice. All values were normalized with respect to the non-transfected cells.

### Western blot analysis

Total proteins were extracted on ice with RIPA lysis buffer supplemented with protease (Sigma-Aldrich; Merck KGaA, #11697498001) and phosphatase (Sigma-Aldrich; Merck KGaA, #4906845001) inhibitors. Fifty micrograms of protein were separated by 10% SDS-PAGE, and transferred to nitrocellulose membranes (GE HealthCare Technologies Inc, IL, USA). Then, membranes were blocked and incubated with primary antibodies for Actin (goat polyclonal antibody; dilution 1:1000; Santa Cruz, #sc-1615) and BORIS (rabbit polyclonal antibody; dilution 1:100; Sigma-Aldrich; Merck KGaA, #Hpa001472), respectively. Membranes were washed and incubated with horseradish peroxidase-conjugated secondary antibodies [anti-rabbit (Santa Cruz, #sc-2357) and anti-goat (Santa Cruz, #sc-2354)] at 1:2500 dilution. The signal was detected using SuperSignal® West Femto Chemiluminescent Substrate (Thermo Fisher Scientific Inc, MA, USA) and images captured on the Fusion Solo S Imaging System (Vilber Lourmat Sté, Collégien, France). BORIS and Actin abundance was determined by standard densitometry analysis, using ImageJ software version 1.53 (National Institutes of Health, MD, USA). Actin was used as normalizing protein.

### Statistical analysis

The R Programming Language version 4.2.2 (R Foundation for Statistical Computing; http://www.R-project.org/) was used in the analysis of Kaplan-Meier curves, DESeq, WGCNA, GO, KEGG, Reactome, and WikiPathways. The Pearson’s correlation coefficient was used to measure the correlation between two continuous variables. The Student´s t-test or Mann-Whitney-Wilcoxon test was performed for comparisons between two groups. The data were presented as mean ± SD and differences were compared via R Programming Language. A *P* value < 0.05 was considered statistically significant.

## Results

### miR-122 is negatively regulated by standard chemotherapy in TNBC cells and is a predictive factor for TNBC patients

**Fig. 1:**
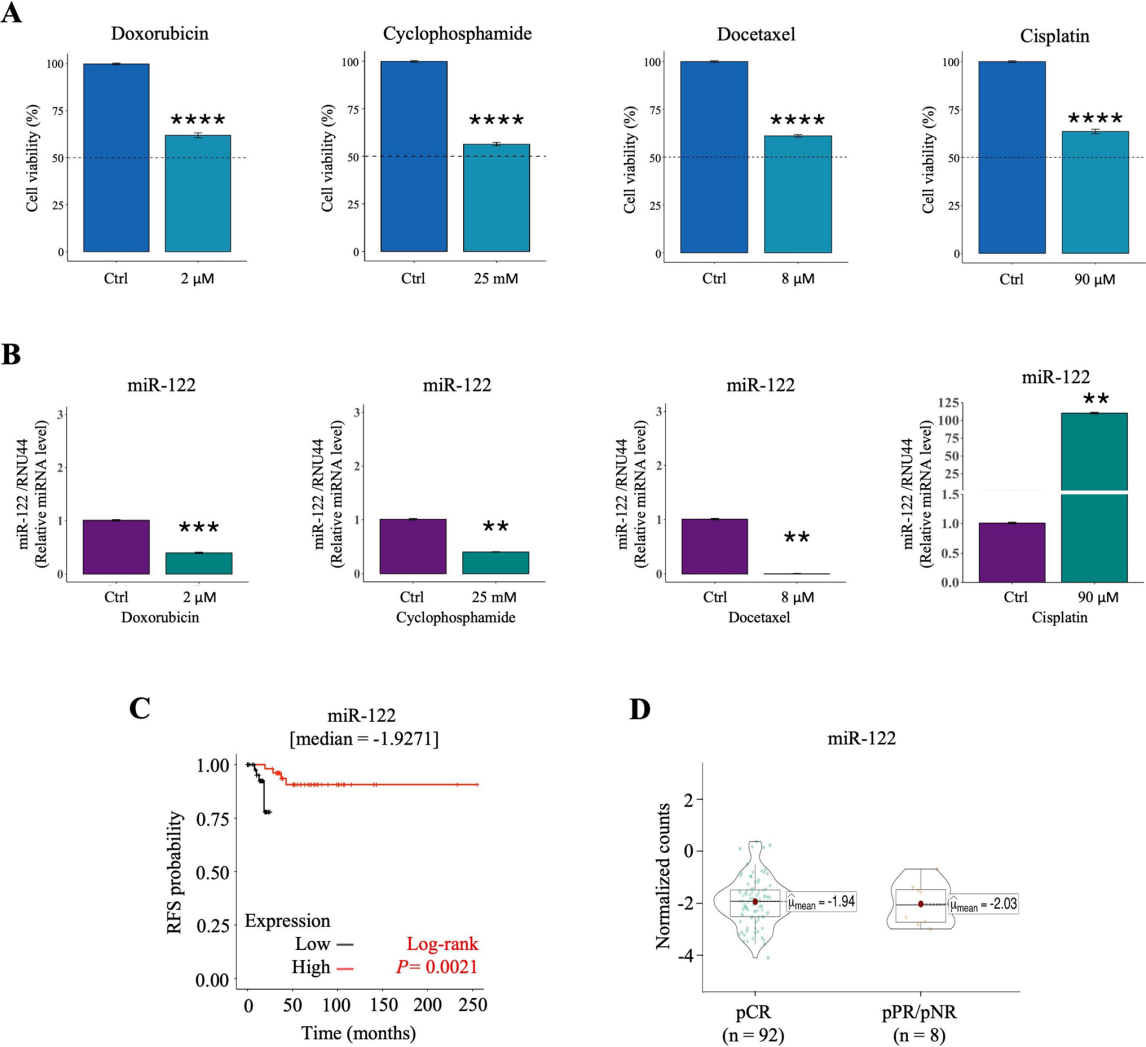
The dysregulation and predictive potential of miR-122 expression to chemotherapy response in TNBC. **A** The MDA-MB-231 cells were treated with IC_50_ of doxorubicin, cyclophosphamide, docetaxel, or cisplatin for 24 h. **B** The expression levels of miR-122 in MDA-MB-231 cells treated with IC_50_ of doxorubicin, cyclophosphamide, docetaxel, or cisplatin for 24 h. **C** Kaplan–Meier plots of RFS in TNBC patients to evaluate the predictive potential of the miR-122 expression. High or low expression levels according to ≥ median or < median of expression, respectively. Data from 105 TNBC cases. *P*-value was calculated by log rank test *P*. **D**The expression analysis of miR-122 in the pathologic response. Red dot in the boxplot indicates the mean of level expression of miR-122. *P ≤ 0.05; **P ≤ 0.01; ***P ≤ 0.001; ****P ≤ 0.0001 by Student´s *t*-test. *P* ≤ 0.05 was considered statistically significant. *TNBC* Triple negative breast cancer, *RFS* Relapsed free-survival, *pCR* Pathological complete response, *pPR* Pathological partial response, *pNR* Pathological non-response

Several studies have revealed that miRNAs are deregulated inside tumor tissues after NAC [40–42], including breast cancer [43–46]. Specifically, has-miR-122-5p (miR-122) levels have been reported to be increased in the serum of TNBC patients after NAC [47]. The taxanes and anthracycline-based regimens represent the cornerstone in TNBC therapy, while platinum-based chemotherapy has shown promising results in the neoadjuvant and metastatic settings [48]. We explore the effect of these drug panel on the expression of miR-122. For this purpose, we used the human TNBC cell line MDA-MB-231. The cells were exposed for 24 h to the half-maximal inhibitory concentration (IC_50_) of doxorubicin, cyclophosphamide, docetaxel, and cisplatin (Supplementary Fig. 1). After that, cell viability (Fig. 1A) and the expression of miR-122 (Fig. 1B) were determined. Interestingly, miR-122 was downregulated by doxorubicin, cyclophosphamide, and docetaxel in MDA-MB-231 cells, whereas cisplatin induced it significant overexpression. These results suggest that at least doxorubicin, cyclophosphamide, and docetaxel could downregulate miR-122 expression to exert their cytotoxic effect. Cisplatin might promote overexpression of miR-122 for better clinical outcomes in TNBC patients and might counteract invasion and metastasis *in vitro* by increasing the miR-122 tumor suppressor. However, this observation must be explored. To better understand the role of miR-122 in TNBC, we evaluated the clinical value of miR-122 expression levels on the treatment response. A total of 105 TNBC patients were used to evaluate recurrence-free survival (RFS) and pathological complete response (pCR) after NAC based on anthracyclines, taxanes, and cyclophosphamide. Most of TNBC patients presented grade II tumors (76 %). Pathological data of estrogen receptor (ER), progesterone receptor (PR) and human epidermal growth factor receptor 2 (HER2) revealed that TNBC subtypes basal like tumors were predominant (92 %). All patient population received neoadjuvant chemotherapy (NAC) between 2010 and 2015. NAC was based on taxanes, anthracyclines, and cyclophosphamide. The mean time to relapse was shorter in miR-122 downregulated population (13.64 ± 7.49 months) compared to miR-122 upregulated population (71.77 ± 47.88 months). There were no significant differences in the clinic-pathological characteristics, treatment scheme or tumor stage of the patients. (Supplementary Table 1). Results showed that low expression of miR-122 was significantly associated with shorter RFS (Fig. 1C). However, miR-122 expression levels were not associated with pathological response (Fig. 1D), highlighting that downregulation of miR-122 was a predictive factor for relapsed in TNBC patients treated with chemotherapy. These results suggested that loss expression of miR-122 might promote a phenotype related to worse outcome after chemotherapy.

### Identification of a transcriptomic profile in tumors with loss of expression of miR-122 of TNBC patients with worse outcome after chemotherapy

**Fig. 2:**
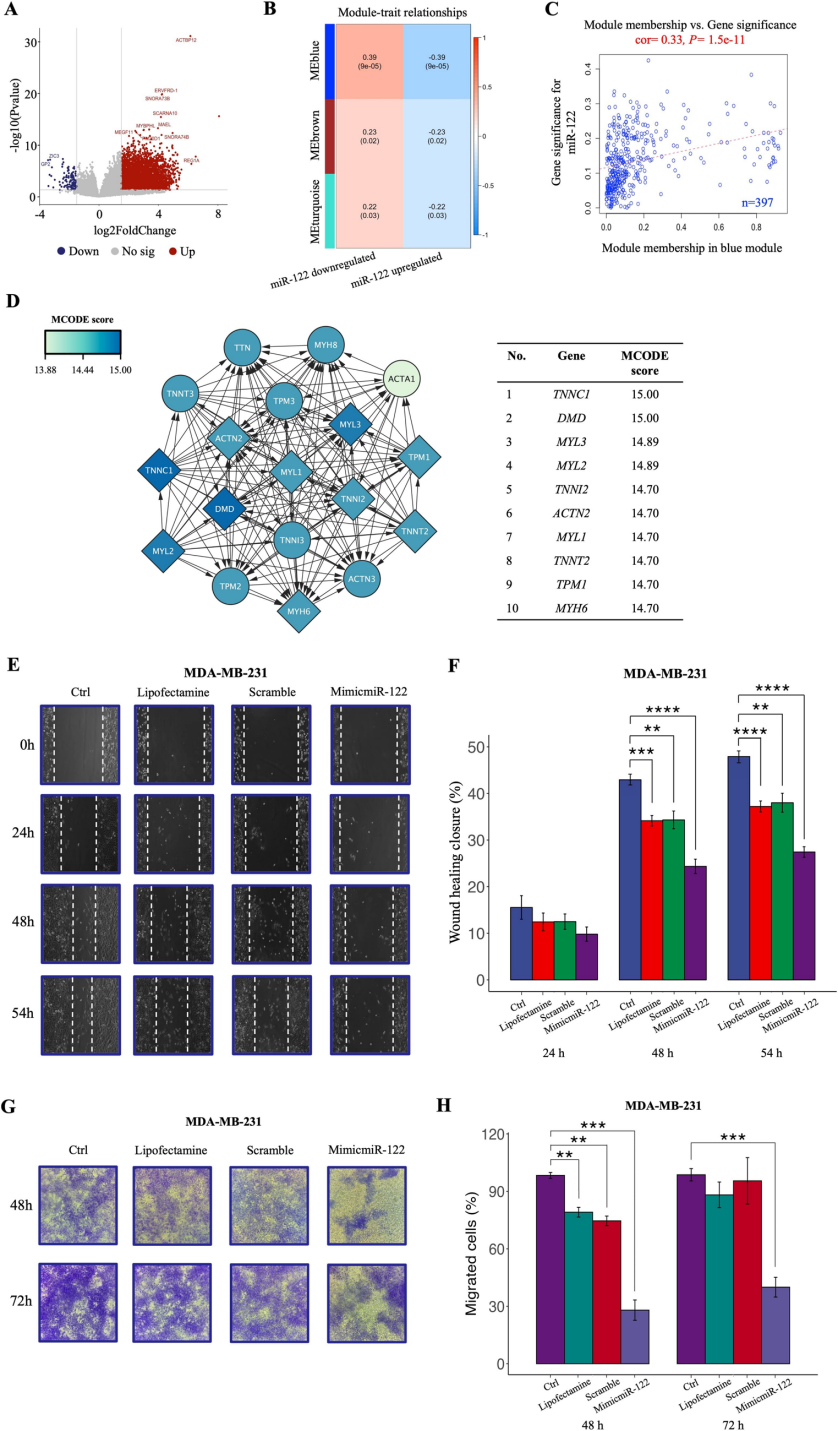
The loss of miR-122 expression and the blue module genes were associated with migration process in TNBC. **A** Volcano plot shows significantly DEGs in TNBC tumors with lower levels of miR-122 expression (designated as miR-122 downregulated) when compared to TNBC tumors with higher levels of miR-122 expression (designated as miR-122 upregulated). **B** Correlation heatmap of the gene modules relative to differential expression of miR-122 by WGCNA. Each cell contains the corresponding coefficient correlation which decreased in size from red (1) to blue (− 1) and *P*-value. **C** Scatter plot of Gene Significance (GS) vs. Module Membership (MM) in the co-expression blue module (n = 397 genes) for the miR-122 downregulated molecular phenotype. cor, Pearson correlation coefficient. **D** MEblue subnetwork based on MCODE score. The diamond-shaped nodes highlight the top ten hub genes from the blue module. The miR-122 overexpression suppressed the migration capacities in MDA-MB-231 cells. Wound healing (**E** and **F**) and transwell (**G** and **H**) assay were perform in MDA-MB-231 with exogenous expression of miR-122. Bars represent the mean ± 1 SD of three independent experiments. **P ≤ 0.01; ***P ≤ 0.001; ****P ≤ 0.0001 by Student´s *t*-test. *P* ≤ 0.05 was considered statistically significant. miR-122, hsa-miR-122-5p; Ctrl, non-transfected cells; MimicmiR-122, hsa-miR-122-5p mimics. *TNBC* Triple negative breast cancer, *DEG* Differential gene expression, *WGCNA* Weighted gene co-expression network analysis

We hypothesized that exists a specific transcriptomic profile and a gene co-expression network associated with short recurrence in TNBC patients treated with NAC and loss of miR-122 expression. The analysis of transcriptome of the TNBC tumors that expressed lower levels of miR-122 (designated as miR-122 downregulated) compared with those that expressed higher levels of miR-122 (designated as miR-122 upregulated) showed 3769 DEGs. Among them, 3679 genes were upregulated, and 90 genes were downregulated (Fig. 2A and Supplementary Table 2). The transcriptomic profile of the 3769 DEGs were transformed into an adjacency matrix, and the co-expression network were constructed by WGCNA. Before network construction and module detection, the 105 TNBC cases were clustered in a sample dendrogram and visualized in a heatmap based on miR-122 downregulated and miR-122 upregulated molecular phenotypes. Because of the presence of outliers, 11 samples (TNBC_13, TNBC_76, TNBC_87, TNBC_89, TNBC_138, and TNBC_179 from miR-122 downregulated; TNBC_12, TNBC_22, TNBC_44, TNBC_97, and TNBC_154 from miR-122 upregulated) were removed for further analysis (Supplementary Fig. 2 A).

The weighted co-expression network was constructed with a soft-threshold *β* = 9 that ensured the adjacency function of the scale-free network (Supplementary Fig. 2B and C). Next, the hierarchical clustering tree was obtained through the correlation coefficient between genes (Supplementary Fig. 2D) and three modules were constructed: MEblue module (n = 397 genes), MEturquoise module (n = 1078 genes), and MEbrown module (n = 2294 genes). Then, the correlation between gene modules and the expression levels of miR-122 was calculated using Pearson’s correlation test analysis method. The blue module was positively correlated with miR-122 downregulated and negatively correlated with miR-122 upregulated (Fig. 2B). While brown and turquoise modules showed lower significance than blue module. Finally, a scatterplot of Gene Significance (GS) vs Module Membership (MM) was applied for the co-expression blue module corroborating that the blue module had a significantly positive correlation (Pearson correlation coefficient= 0.33, *P* = 1.5e-11) with the loss of miR-122 expression (Fig. 2C). Therefore, the blue module was identified as significant co-expressed genes module for tumors with loss of expression of miR-122 of the TNBC patients with poor outcome (Supplementary Table 3), compared with those expressed higher levels of miR-122. Thus, the co-expressed genes from the blue module were considered for further analysis.

### The co-expressed genes by loss of expression of miR-122 are functionally enriched in cell differentiation, tumor growth and metastasis pathways

To link the biological function of the co-expressed genes from the blue module with miR-122 downregulation in TNBC we constructed the protein-protein interaction (PPI) network, as well as the enrichment analysis was performed through GO enrichment, KEGG pathways, Reactome pathways, and WikiPathways. Overall, co-expressed genes from the blue module are implicated in cell differentiation, cell communication and signaling, and cytoskeleton organization (Supplementary Fig. 3 and Supplementary Fig. 4 A). Some genes are also related to extracellular exosomes, chromatin organization and epigenetics (Supplementary Fig. 4B–D).

The co-expressed genes from the blue module were screened out in PPI subnetwork to identify genes with higher connectivity via Molecular Complex Detection (MCODE) score using Cytoscape. A highly interconnected PPI subnetwork with 18 nodes, 150 edges and confidence score of 17.65 was obtained. *TNNC1*, *DMD*, *MYL3*, *MYL2*, *TNNI2*, *ACTN2*, *MYL1*, *TNNT2*, *TPM1*, and *MYH6* genes were found as top ten hub genes in the co-expressed genes by the loss of miR-122 expression (Fig. 2D). Remarkably, hub genes coding to proteins associated with actin cytoskeleton, probably for promote motility and contraction of the cancer cells, which are processes highly related to metastasis and disease progression or recurrence. Taken together, all these results suggested that miR-122 downregulation promotes transcriptional changes that favor cell differentiation, cell communication, tumor growth, motility, and metastasis. Based on these results, we evaluated whether miR-122 expression influences the cellular migration of TNBC cells. Cell migration was evaluated by wound healing and transwell assays in MDA-MB-231 cells with exogenous overexpression of miR-122 (Supplementary Fig. 5). Results showed that at 30, 48 and 54 h after wounding, the healing ability of miR-122-transfected MDA-MB-231 cells significantly lagged up to 20% behind the non-transfected and transfection control cells (Fig. 2E and F). The above results were confirmed by the transwell assay in which inhibition in the migration of miR-122-transfected MDA-MB-231 cells compared to non-transfected and transfection control cells was observed at 48 and 72 h (Fig. 2G and H), suggesting that miR-122 could inhibit cell migration. In overall, the results showed that loss of miR-122 expression in TNBC could promote a migratory phenotype.

### Co-expressed genes by loss of miR-122 expression are implicated in advanced stage, worse prognosis, and recurrence in TNBC patients

**Fig. 3:**
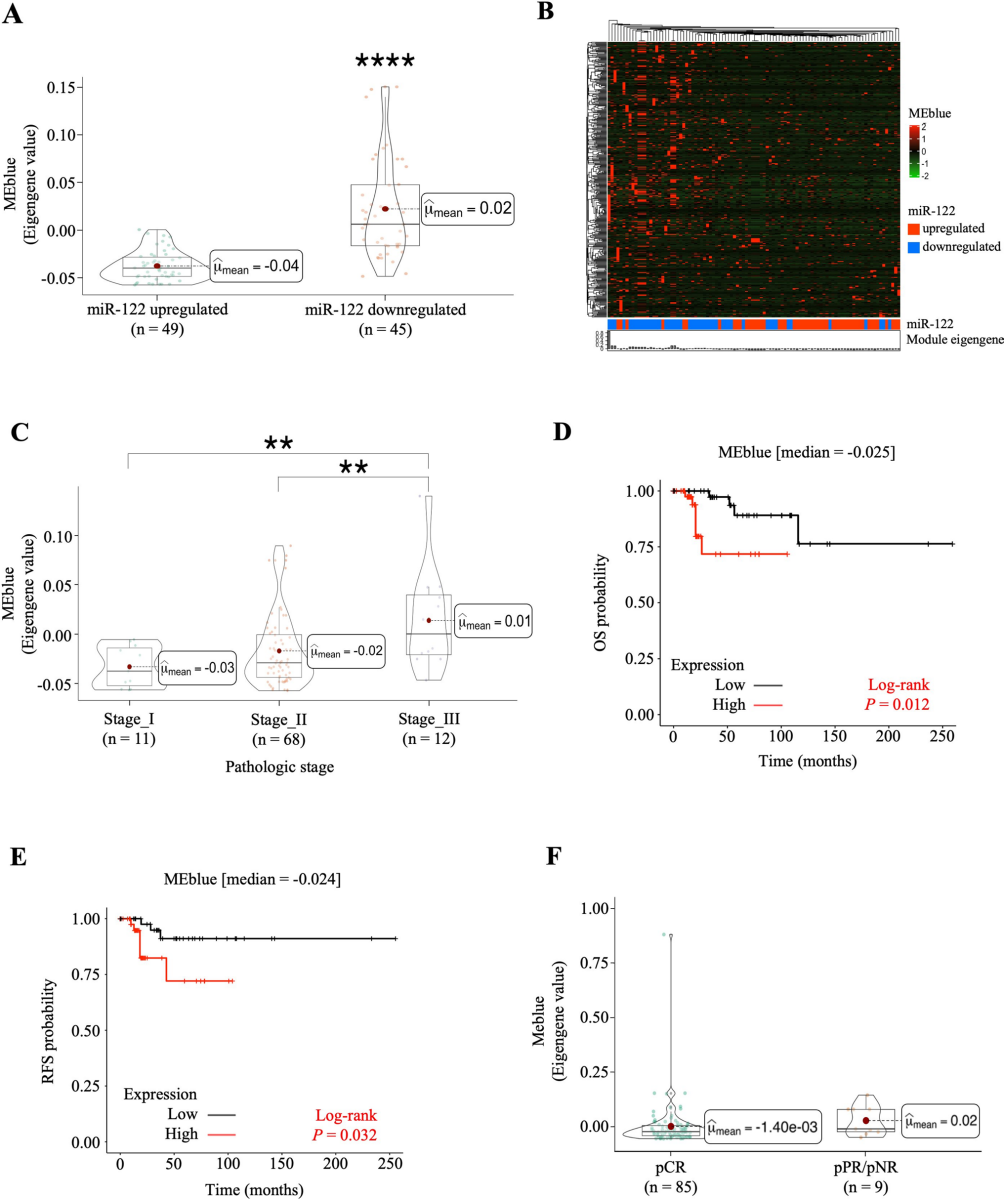
The expression of blue module genes was associated with advanced stage, poorest OS and shorter RFS in TNBC. **A** Box plot and **B** heat map shows the differences in the blue module expression between miR-122 downregulated molecular phenotype with respect to miR-122 upregulated molecular phenotype. **C** Box plot of blue module expression between pathologic stages in TNBC patients. Red dot in the boxplot indicates the mean of level expression of blue module. Kaplan–Meier plots of **D** OS and **E** RFS in TNBC patients to evaluate the prognostic and predictive potential of the blue module expression. High or low expression levels according to ≥ median or < median of blue module expression, respectively. The Kaplan–Meier curves were compared using a log rank test *P*. **F** The expression analysis of blue module in the pathologic response. Red dot in the boxplot indicates the mean of level expression of blue module. **P ≤ 0.01; ****P ≤ 0.0001 by Wilcoxon test. *P* ≤ 0.05 was considered statistically significant. *pCR* Pathological complete response, *pPR* Pathological partial response, *pNR* Pathological non-response, *TNBC* Triple negative breast cancer, *OS* Overall survival, *RFS* Relapsed free-survival, *pCR* Pathological complete response, *pPR* Pathological partial response, *pNR* Pathological non-response

To ensure the reliability of the results for co-expressed genes and its correlation with loss of miR-122 expression, the set of co-expressed genes was analyzed by calculating the eigengenes for all TNBC population and to explore its clinical value in TNBC patients. First, the eigengene distribution was evaluated in TNBC cases according to gain or loss of miR-122. The results showed that the eigenvalue of the blue module was found to have a high correlation with loss of expression of miR-122 because the set of co-expressed genes was upregulated in tumors with downregulated miR-122 when compared to tumors with upregulated miR-122 (Fig. 3A). Moreover, the hierarchical clustering was performed by dividing the patients according to gain or loss of miR-122 to determine the degree of genes dysregulation for each patient. Interestingly, the expression of the blue module was able to discriminate between loss and gain of miR-122 and an activation pattern of the set of genes was observed in tumor samples with loss of miR-122 expression (Fig. 3B).

Interestingly, the expression of blue module between stages of tumors showed high correlation to the TNBC tumors in stage III which had significantly higher eigengene values (Fig. 3C), indicating that the activation of the blue module was more related to advanced stage in TNBC. To determine whether the expression of blue module could be a prognostic and predictive factor in TNBC, the overall survival (OS) and relapse-free survival (RFS) analyses were performed. According to the Kaplan-Meier curves, high expression of blue module was significantly associated with poorest OS (Fig. 3D) and shorter RFS (Fig. 3E) in TNBC patients. However, blue module expression levels were not associated with pCR in TNBC patients (Fig. 3F). Taken together, these results showed that loss of miR-122 expression allowed the co-expression activation of 397 genes (giving rise to the blue module) associated to advanced stage, worse prognosis and recurrence in TNBC patients under taxanes and anthracycline-based treatment.

### Blue module contains target genes of miR-122

**Fig. 4:**
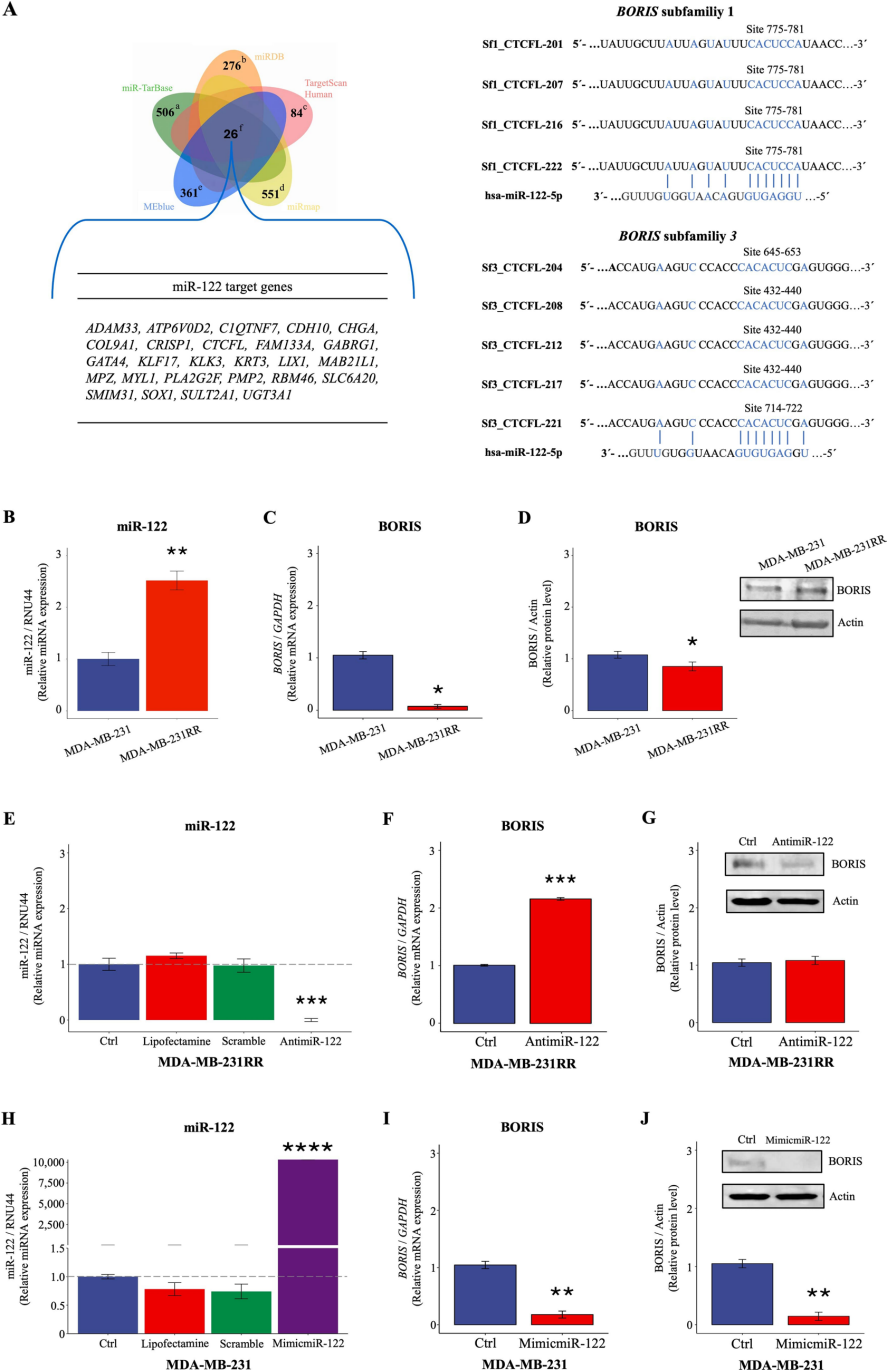
BORIS is regulated by miR-122 in TNBC cells. **A** A Venn diagram depicting miR-122 target genes and in silico analysis of the seed-region hsa-miR-122-5p binding sites on 3´-UTR of the *BORIS* subfamily 1 and subfamily 3. Letters in Venn diagram indicate the miR-122 target genes identified in miR-Tar base (**a**), miRDB (**b**), TargetScan Human (**c**), miRmap (**d**), and the co-expressed gene module MEblue (**e**). In total, among the four prediction web tools, 26 miR-122 target genes (**f**) were identified in the MEblue module. **B** Endogenous expression of miR-122 in MDA-MB-231 and MDA-MB-231RR cells. Endogenous levels of *BORIS* **C** mRNA and **D** protein in MDA-MB-231 and MDA-MB-231RR cells. **E** The knockdown of miR-122 expression in MDA-MB-231RR cells after transfection with AntimiR-122 was confirmed by RT-qPCR. Levels of *BORIS* **F** mRNA and **G** protein in MDA-MB-231RR cells transfected with AntimiR-122. **H** The overexpression of miR-122 in MDA-MB-231 cells after transfection with MimicmiR-122 was confirmed by RT-qPCR. Levels of *BORIS* **I** mRNA and **J** protein in MDA-MB-231 cells transfected with MimicmiR-122. All results were compared with non-transfected control cells (Ctrl) and transfection control cells (Lipofectamine and Scramble). Bars represent the mean ± 1 SD of three independent experiments. *P ≤ 0.05; **P ≤ 0.01; ***P ≤ 0.001; ****P ≤ 0.0001 by Student´s *t*-test. *P* ≤ 0.05 was considered statistically significant. miR-122, hsa-miR-122-5p; Ctrl, non-transfected cells; AntimiR-122, hsa-miR-122-5p inhibitor; MimicmiR-122, hsa-miR-122-5p mimics. *TNBC* Triple negative breast cancer, *UTR* Untranslated region

According to bioinformatic prediction, we identified 26 potential target genes for miR-122 in the blue module (Fig. 4A). Genes were involved in biological processes related to sustaining proliferative signaling and enabling replicative immortality hallmarks, epigenetic process, immune response, and drug response (Table 1). Ten genes were associated with pPR/pNR after NAC. Specifically, the upregulation of *KLK3*, *MYL1*, *CHGA*, *GATA4*, *PLA2G2F, CRISP1* and *SULT2A1* genes and downregulation of *LIX1, ADAM33,* and *FAM133A* gene were related to chemotherapy failure (Supplementary Fig. 6). On the other hand, the Kaplan-Meier analysis revealed that the low expression of *SOX1*, *UGT3A1, LIX1, GATA4*, and *CTCFL* genes, and the high expression of *CHGA*, *MPZ*, *PLA2G2F*, *CDH10*, *COL9A1*, and *MAB21L1* genes were associated to shorter RFS (log-rank test, *P* < 0.05) (Supplementary Fig. 7). All these findings revealed target genes of miR-122 as predictive factors for long-term clinical outcome in TNBC patients.

**Table 1:** The miR-122 target genes in the blue module

No	Symbol	Gene name	log2 FC	Start (nt)	End (nt)	Binding region length (nt)	Position	Biological processes
1	*SOX1*	SRY-Box Transcription Factor 1	4.18	2149	2187	38	3’*UTR*	Sustaining proliferative signaling and Enabling Replicative Immortality Hallmarks
2	*UGT3A1*	UDP Glycosyltransferase Family 3 Member A1	2.76	1451	1471	20	3’*UTR*	Xenobiotic metabolism, tamoxifen metabolism, and drug metabolism
3	*KLK3*	Kallikrein Related Peptidase 3	2.67	1244	1267	23	3’*UTR*	Sustaining proliferative signaling and Enabling Replicative Immortality Hallmarks
4	*MYL1*	Myosin Light Chain 1	2.5	772	796	24	3’*UTR*	Cell cycle
5	*LIX1*	Limb and CNS expressed 1	2.19	2786	2801	15	3’*UTR*	Sustaining proliferative signaling and Enabling Replicative Immortality Hallmarks
6	*CHGA*	Chromogranin A	2.19	1735	1755	20	3’*UTR*	Innate immune response
				1282	1302	20	3’*UTR*	
7	*GABRG1*	Gamma-Aminobutyric Acid Type A Receptor Subunit Gamma1	2.08	5209	5232	23	3’*UTR*	Sodium ion transmembrane transport, lipid transport, and calcium ion transmembrane transport
8	*MPZ*	Myelin protein zero	2.04	1427	1448	21	3’*UTR*	Cell cycle
9	*GATA4*	GATA Binding Protein 4	2.02	2698	2716	15	3’*UTR*	Cell differentiation, Wnt signaling, cancer cell stemness, and positive regulation of cell proliferation
				1910	1937	15	3’*UTR*	
				2695	2713	15	3’*UTR*	
10	*RBM46*	RNA binding motif protein 46	2	1847	1880	16	3’*UTR*	Cell differentiation, Wnt signaling, cancer cell stemness, and positive regulation of cell proliferation
				1790	1823	16	3’*UTR*	
11	*C1QTNF7*	C1q and TNF related 7	1.99	1500	1517	17	3’*UTR*	Cell cycle
12	*PLA2G2F*	Phospholipase A2 Group IIF	1.88	2113	2132	19	3’*UTR*	Sustaining proliferative signaling and Enabling Replicative Immortality Hallmarks
13	*CTCFL*	CCCTC-binding factor like	1.85	2920	2938	18	3’*UTR*	DNA methylation, histone modification, and chromatin remodeling
				3392	3410	18	3’*UTR*	
				2797	2816	19	3’*UTR*	
				3216	3234	18	3’*UTR*	
				2848	2867	19	3’*UTR*	
				2837	2856	19	3’*UTR*	
				2177	2196	19	3’*UTR*	
				2050	2069	19	3’*UTR*	
				3020	3038	18	3’*UTR*	
14	*CRISP1*	Cysteine Rich Secretory Protein 1	1.83	1750	1769	19	3’*UTR*	Calcium channel regulator activity
				1825	1858	20	3’*UTR*	
15	*PMP2*	Peripheral myelin protein 2	1.79	3396	3413	17	3’*UTR*	Sodium ion transmembrane transport, lipid transport, and calcium ion transmembrane transport
				1082	1109	27	3’*UTR*	
				3223	3240	17	3’*UTR*	
16	*SMIM31*	Small Integral Membrane Protein 31	1.77	3070	3094	18	3’*UTR*	Cell differentiation, Wnt signaling, cancer cell stemness, and positive regulation of cell proliferation
17	*ADAM33*	ADAM metallopeptidase domain 33	1.76	2645	2667	22	3’*UTR*	Cell differentiation, Wnt signaling, cancer cell stemness, and positive regulation of cell proliferation
				2570	2592	22	3’*UTR*	
				2648	2670	22	3’*UTR*	
18	*FAM133A*	Family with sequence similarity 133 member A	1.74	1247	1265	18	3’*UTR*	Cell cycle
				1178	1196	18	3’*UTR*	
19	*SLC6A20*	Solute carrier family 6 member 20	1.72	5031	5063	32	3’*UTR*	Sodium ion transmembrane transport, lipid transport, and calcium ion transmembrane transport
				4718	4738	20	3’*UTR*	
				4920	4952	32	3’*UTR*	
				4607	4627	20	3’*UTR*	
20	*KRT3*	Keratin 3	1.68	2037	2056	19	3’*UTR*	Cell differentiation, Wnt signaling, cancer cell stemness, and positive regulation of cell proliferation
21	*ATP6V0D2*	ATPase H + transporting V0 subunit d2	1.65	2053	2075	22	3’*UTR*	Cell differentiation, Wnt signaling, cancer cell stemness, and positive regulation of cell proliferation
22	*KLF17*	Kruppel like factor 17	1.64	2470	2518	17	3’*UTR*	Cell cycle
23	*SULT2A1*	Sulfotransferase family 2 A member 1	1.62	1457	1481	18	3’*UTR*	Xenobiotic metabolism, tamoxifen metabolism, and drug metabolism
24	*CDH10*	Cadherin 10	1.58	2916	2938	22	3’*UTR*	Sustaining proliferative signaling and Enabling Replicative Immortality Hallmarks
				2555	2577	22	3’*UTR*	
25	*COL9A1*	Collagen type IX alpha 1 chain	1.56	2430	2462	32	3’*UTR*	Cell cycle
				3174	3206	32	3’*UTR*	
26	*MAB21L1*	Mab-21 like 1	1.55	1891	1908	17	3’*UTR*	Cell differentiation, Wnt signaling, cancer cell stemness, and positive regulation of cell proliferation

### miR-122 regulates the expression of *BORIS* in TNBC cells

We found that *BORIS* contains one binding site for miR-122 conserved in the 3´-UTR of 9 mRNA isoforms categorized as subfamilies 1 and 3 (Fig. 4A). Thus, we explore whether BORIS could be regulated by miR-122. For this purpose, we used two TNBC cell lines (MDA-MB-231 and MDA-MB-231RR) that express different levels of miR-122 [21] as an *in vitro* model of miR-122 dysregulation. We previously reported that parental MDA-MB-231 cells express low levels of miR-122 with respect to MDA-MB-231RR cells [21]. We evaluated the endogenous expression of miR-122 (Fig. 4B) and *BORIS* (Fig. 4C and D) in MDA-MB-231 and MDA-MB-231RR cells. The results showed that miR-122 was overexpressed in MDA-MB-231RR cells than MDA-MB-231 cells (Fig. 4B) as previously were reported [21]. While *BORIS* expression analysis showed lower levels of mRNA (Fig. 4C) and protein (Fig. 4D) in MDA-MB-231RR cells than MDA-MB-231 cells. We proved the inverse relationship among miR-122 and *BORIS* by compared their expression levels in TNBC cells with respect non-tumorigenic human breast MCF-10A cells (Supplementary Fig. 8). These results showed an inverse relationship between the miR-122 expression and *BORIS* gene expression in TNBC cells suggesting that *BORIS* gene could be regulated by miR-122. To validate if the expression of BORIS could be regulated by miR-122, we blocked or overexpressed miR-122 in MDA-MB-231RR and MDA-MB-231 cells, respectively. Before evaluating the effect of miR-122 on BORIS, the knockdown and exogenous overexpression of miR-122 were confirmed (Fig. 4E–H). Remarkably, we observed that knockdown of miR-122 successfully increased *BORIS* expression at mRNA level (Fig. 4F) but no change was observed at protein level (Fig. 4G) in MDA-MB-231RR cells, suggesting that the increase of mRNA of *BORIS* is not enough to increase protein levels, which may occur through another mechanism in the translational control. In contrast, the exogenous upregulation of miR-122 successfully suppressed *BORIS* gene at both mRNA (Fig. 4I) and protein level (Fig. 4J) within MDA-MB-231 cells. Thus, we confirmed that *BORIS* gene could be regulated by miR-122 in TNBC cells.

### *BORIS* overexpression is associated to transcriptome enriched in metastasis and invasion genes in TNBC tumors

**Fig. 5:**
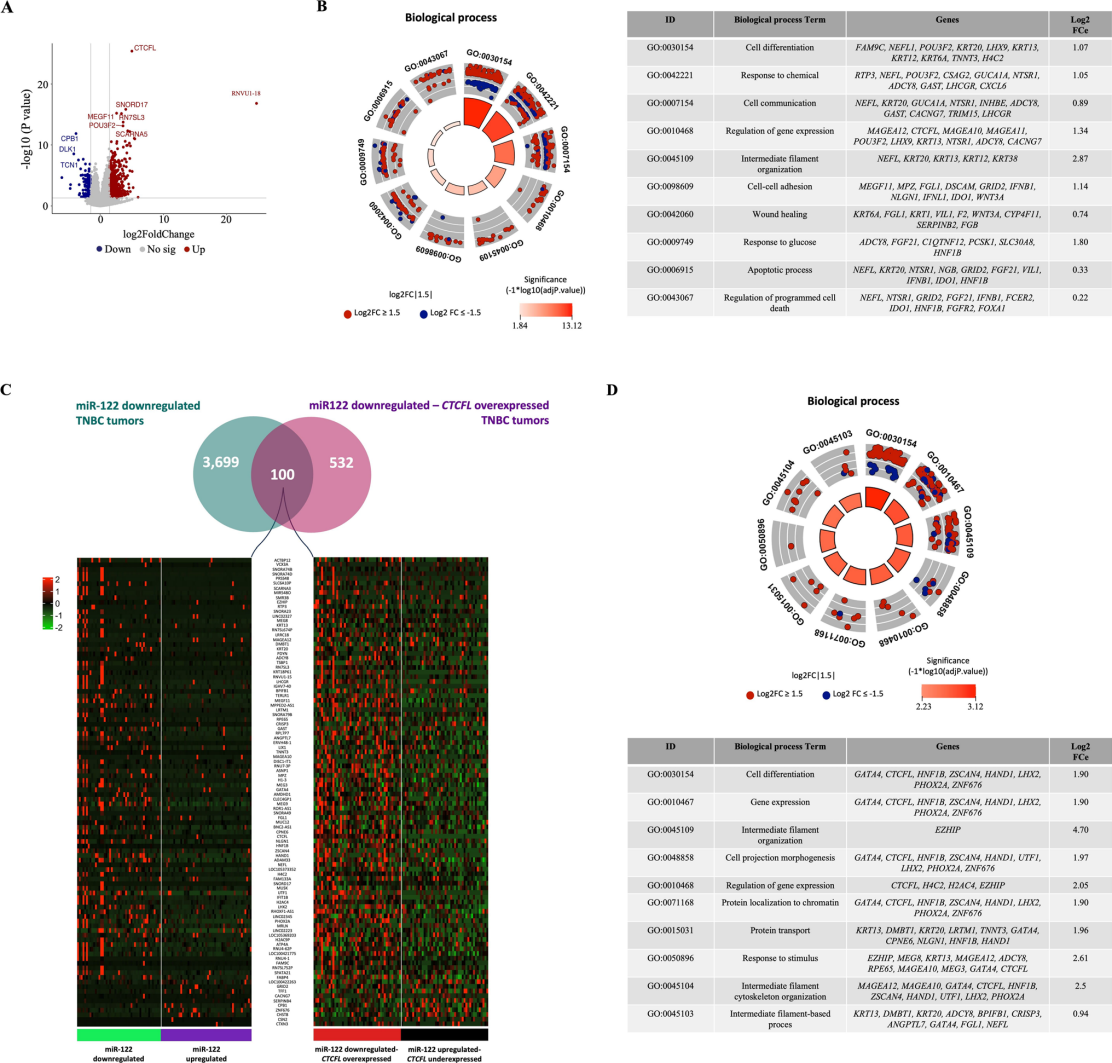
High *CTCFL* expression in TNBC tumors with loss of miR-122 expression displayed a transcriptomic profile related to invasion and migration. **A** Volcano plot shows significantly DEGs in TNBC tumors expressed lower levels of miR-122 and higher levels of *CTCFL*. **B** Circular plot of gene ontology (GO) analysis based on biological process from DEGs in *CTCFL*-overexpressing TNBC tumors (n = 632 genes). **C** Venn diagram and heatmap showing genes shared between the transcriptome of miR-122 downregulated TNBC tumors and transcriptome of miR-122 downregulated-*CTCFL* overexpressed TNBC tumors. **D** Circular plot of GO analysis based on biological process from genes shared between miR-122 downregulated phenotype and miR-122 downregulated-*CTCFL* overexpressed phenotype TNBC tumors (n = 100 genes). *TNBC* Triple negative breast cancer, *GO* Gene ontology

BORIS is a chromatin remodeler that is overexpressed in TNBC tumors with low levels of miR-122, therefore we sought to identify the transcriptomic profile of TNBC tumors simultaneously expressing lower levels of miR-122 and overexpressing *BORIS*. We used the data-seq from TNBC tumors used in the transcriptome analysis of miR-122 obtained from the TCGA-BRCA project. We identified 84 downregulated and 548 upregulated genes (Fig. 5A and Supplementary Table 4). DEGs were related to cell differentiation and communication, cytoskeletal components including actin-binding proteins, actin mediated cell contraction, extracellular matrix, and extracellular exosomes (Fig. 5B and Supplementary Fig. 9). These results were coincident with the transcriptome promoted by loss expression of miR-122. One hundred of these DEGs, are conserved in all tumors with loss expression of miR-122 (Fig. 5C and Supplementary Table 5), including *BORIS* overexpression (Fig. 5A). These genes were also related to regulation of gene expression, protein associated to chromatin and cytoskeleton function (Fig. 5D). All these results suggest an autoregulation of *BORIS* in TNBC tumors with loss expression of miR-122 to maintain a migratory phenotype through its function as transcriptional modulator. The role of axis miR-122-BORIS on the rapid-relapsed of the TNBC patients submitted to chemotherapy could be related to the control of cell migration and invasion throughout regulation of cytoskeletal, cell differentiation or cell communication genes. This hypothesis must be proved.

## Discussion

In this work, we aimed described the role of miR-122 in TNBC under chemotherapy treatment. TNBC is a subtype of breast cancer highly heterogeneous [49, 50]. Chemotherapy is currently the major systemic treatment for TNBC, but unfortunately, patients often develop resistance to chemotherapy and metastasis leading to poor prognosis and mortality. Tumor suppressor miRNAs are promising candidates for developing cancer treatments due to their regulatory capacity, which permits modulation of whole signaling pathways inside the cells [7, 51]. Currently miR-122 is considered as a tumor suppressor in breast cancer [16], including TNBC [52]; however, miR-122 may function as oncomiR in breast cancer cells resistant to radiotherapy [21]. In addition, it has been shown that miR-122 may also play a role in the chemotherapy response in cancer [10–12], including breast cancer [13, 14]. These findings suggest that miR-122 may have different oncogenic functions in breast cancer. However, there is still no related evidence of the functional mechanism of miR-122 as tumor suppressor in TNBC, mainly related to chemotherapy response. Intensive research efforts have deduced that miRNAs have a dual function in cancer, particularly in TNBC, as tumor suppressors or oncogenes (oncomiRs). Currently, it is recognized that the dual role of miRNAs, as a tumor suppressor or oncomiR, is determined by the affinity to their target mRNA and the interaction of the RNA-binding proteins (RBPs) present in the 3´-UTR on the same target mRNA [53]. Therefore, the combinatorial effect between RBPs and miRNAs promotes that a single miRNA can have a dual role in carcinogenesis, achieving a highly refined modulation of the expression of genes. Specifically, it is recognized that miR-122 is aberrantly expressed in various tumors, including breast cancer, acting as both a oncomiR and tumor suppressor. Perez-Añorve et al. (2019) revealed that miR-122 has a role as a tumor suppressor in parental breast cancer cells by decreasing survival and promoting radiosensitivity. However, in radioresistant breast cancer cells, miR-122 functions as an oncomiR by promoting survival [21]. Moreover, the role of extracellular miR-122 in the development and metastasis of breast cancer was studied by Fong et al. (2015). They show that by secreting vesicles with elevated amounts of miR-122, cancer cells may stop non-tumor cells in the pre-metastatic niche from taking glucose [54]. However, miR-122 has also been described as oncomiR in TNBC. Wang et al. (2020) found that when comparing TNBC cells to normal cell lines, miR-122 was considerably elevated, and the CHMP3 gene dramatically downregulated. Additionally, upregulating the CHMP3 gene had the opposite effect from miR-122-5p mimics, which increased TNBC cell survival, proliferation, and invasion. They concluded that miR-122, via inhibiting CHMP3 through MAPK signaling, increases aggressiveness and EMT in TNBC [20]. In summary, there is evidence demonstrating the dual role of miR-122 as an oncogenic miRNA or tumor suppressor, supporting the idea that miR-122 deregulation is a crucial component in the growth and progression of malignancies.

We showed that lower levels of miR-122 were associated with worse long-term clinical outcome after NAC in TNBC patients. It is important to highlight that miR-122 expression was able to be a predictive factor for RFS after NAC in TNBC patients, but not for pCR, suggesting that loss of miR-122 expression promotes a change in the transcriptome that could favor biological processes associated with cancer advantages in TNBC tumors, such as response tumor growth, differentiation, motility, and metastasis [55–57].

Indeed, we identified DEGs and co-expressed genes network associated to miR-122 downregulated and worse clinical outcome relative to TNBC samples with higher expression of miR-122. Our results demonstrated that loss of miR-122 expression allowed the co-expression activation of 397 genes (giving rise to the blue module). We identified *TNNC1*, *DMD*, *MYL3*, *MYL2*, *TNNI2*, *ACTN2*, *MYL1*, *TNNT2*, *TPM1*, and *MYH6* as genes highly connected in transcriptome related to loss of miR-122. Hub genes are involved in actin-mediated cell contraction, cell motility, and invasion potential via cytoskeleton reorganization, which have been reported as promotors of cancer progression, metastasis, and resistance to chemotherapy in different types of cancer [58–61]. Functionally, we demonstrate that miR-122 inhibited the cell migration of TNBC cells. Overall, our results showed that the loss of expression of miR-122 increases cell migration and consequently increases the risk of recurrence in TNBC patients.

Notably, the genes co-expression network relative to loss of miR-122 was positively associated with advanced stage of the disease and was negatively associated with OS and RFS in patients with TNBC. However, this group of genes were no associated to pCR. This observation is relevant because this set of co-expressed genes could represent a predictive gene signature for the clinical outcome after NAC in TNBC patients. Particularly, to delimit the role of miR-122, we identified 26 potential miR-122 target genes into the co-expressed genes, of which eleven were associated to clinical outcome after NAC in TNBC patients. *BORIS* was identified as putative target of miR-122. *BORIS* has been implicated in transcriptional switch to promote the invasive phenotype in melanoma [62]; in addition, the chemo-response of breast cancer cells by effective apoptosis has been associated with overexpression of *BORIS* [63]. Currently, *BORIS* is recognized to encode 23 transcript variants, which are grouped into 6 isoforms families [64]. The bioinformatics prediction showed that miR-122 could regulated 9 mRNA isoforms of BORIS categorized as subfamilies 1 and 3 [64]. We demonstrated a negative correlation between the endogenous expressions of miR-122 and *BORIS* in TNBC cell lines. In addition, the exogenous overexpression or knockdown of miR-122 resulted in modulation of *BORIS* expression. Thus, we demonstrated for the first time that *BORIS* is regulated by miR-122 in TNBC. This is the first report showing regulation of *BORIS* expression by miRNAs in cancer. Overexpression of *BORIS* was associated with migration processes, which coincided with the promotion of cell migration by loss of miR-122. BORIS is a paralog of the multifunctional *CTCF* gene [30], and under physiological circumstances, BORIS is expressed in embryonic stem cells and testis for spermatogenesis [24, 64–66]. Currently, the aberrantly BORIS expression has previously been identified in numerous cell lines and primary human cancers of diverse histological origin [24, 67–69], including breast cancer [70]. BORIS has also been proposed to play a role in tumorigenesis [71] and invasive phenotype [62]. In this context, our results demonstrated that high expression of *BORIS* gene was associated to better RFS in TNBC patients under taxanes and anthracycline-based treatment obtained from two independent databases (KM-plotter and TCGA-BRCA datasets). However, whether BORIS expression is dysregulated after chemotherapy treatment in TNBC cells is currently undetermined. Additionally, we identified 632 DEGs in CTCFL-overexpressing TNBC tumors with loss expression of miR-122, which were associated with processes of cell communication and differentiation, apoptosis, and cellular migration and invasion, suggesting that BORIS overexpression could contributes to a metastatic state at the transcriptional level in TNBC. Our finding is also consistent with previous findings by Janssen et. al. whose demonstrated that inducible BORIS overexpression in melanoma cells reduced proliferation and increased migration and invasion, demonstrating that the transcriptional reprogramming by BORIS might be accompanied by a phenotypic switch [62].

Finally, there are still some limitations to our research. First, the data used in this study were based on publicly available datasets without validating in prospective clinical trials. The isoforms of BORIS expressed in TNBC must be identified. The functional validation of BORIS as target of miR-122 by luciferase assays must be demonstrated in order to identify the isoforms of BORIS regulated by miR-122. However, our information is a valuable starting point for follow-up experiments of the mechanistic study of miR-122-BORIS axis.

## Conclusion

We showed that loss of the tumor suppressor miR-122 was associated to rapid-relapsed after NAC in TNBC patients. Loss expression of miR-122 allows upregulation of gene expression profile signature related to metastasis and worse outcome in TNBC patients. Functionally, miR-122 regulates migration and expression of DNA binding protein BORIS. To our knowledge, this is the first report of the regulation of BORIS by a microRNA.

## Supplementary Information

Below is the link to the electronic supplementary material.

Supplementary file1 (DOCX 16 KB)

Supplementary file2 (XLS 915 KB)

Supplementary file3 (XLS 135 KB)

Supplementary file4 (XLS 174 KB)

Supplementary file5 (XLSX 29 KB)

Supplementary Fig. 1. IC50 for drug treatment panel in TNBC cells. Determination of IC50 of (A) doxorubicin, (B) cyclophosphamide, (C) docetaxel and (D) cisplatin in MDA-MB-231 cells treated for 24 hours. The bars represent the average of 3 biological replicates ±1 SD. **P ≤ 0.01, ***P ≤ 0.001, ****P ≤ 0.0001 by Student´s t-test. P ≤ 0.05 was considered statistically significant. Supplementary file6 (JPG 227 KB)

Supplementary Fig. 2. Weighted gene co-expression network construction from miR-122 downregulated and miR-122 upregulated clinical phenotypes. (A) Sample clustering analysis to detect outliers based on the TCGA dataset and heatmap based on molecular phenotypes (miR-122 downregulated in blue and miR-122 upregulated in red). (B) Relationship between scale-free topology model fit and soft-thresholds (powers). (C) Relationship between the mean connectivity and soft-thresholds (powers). (D) Dendrogram of the genes modules based on a dissimilarity measure. miR-122, hsa-miR-122-5p; cor, Pearson correlation coefficient. P ≤ 0.05 was considered statistically significant. Supplementary file7 (TIFF 18006 KB)

Supplementary Figure 3. Functional enrichment network of blue module. Functional enrichment network analysis from the blue module. The network represents the 25% of 247 genes. Functional enrichment based on Gene Ontology, KEGG pathways, Reactome pathways and WikiPathways categories. Supplementary file8 (JPG 418 KB)

Supplementary Figure 4. Functional enrichment analysis of blue module. Bar plots of gene ontology (GO) analysis from the blue module based on (A) biological process, (B) cellular component, (C) molecular function, and significantly enrichment (D) KEGG pathways from blue module (n= 397 genes). Each bar contains the top 10 genes based on the adjusted P value (to the right of the bar) that enrich the pathway. P ≤ 0.05 was considered statistically significant. Supplementary file9 (JPG 349 KB)

Supplementary Figure 5. Upregulation of miR-122 impairs the migration capabilities of TNBC cells. (A-C) The miR-122 overexpression suppressed wound healing in MDA-MB-231 cells at 30 h, 48 h, and 54 h. The results were compared with control (Ctrl) cells and transfection control cells (Lipofectamine and Scramble). Bars represent the mean ±1 SD of three independent experiments. **P ≤ 0.01; ***P ≤ 0.001; ****P ≤ 0.0001 by Student´s t-test. P ≤ 0.05 was considered statistically significant. Ctrl, non-transfected cells; MimicmiR-122, hsa-miR-122-5p mimics. Supplementary file10 (JPG 651 KB)

Supplementary Figure 6. The miR-122 target genes were associated to pCR. The expression analysis of the miR-122 target genes in TNBC patients. Red dot in the boxplot indicates the mean of level expression of each gene. *P ≤ 0.05; **P ≤ 0.01 by Wilcoxon test. P ≤ 0.05 was considered statistically significant. pCR, pathological complete response; pPR, pathological partial response; pNR, pathological non-response. Supplementary file11 (JPG 1056 KB)

Supplementary Figure 7. The miR-122 target genes predict RFS survival. Kaplan-Meier plots of RFS in TNBC patients to evaluate the predictive potential of the expression of miR-122 target genes. High or low expression levels according to ≥ median or <median of gene expression respectively. The Kaplan-Meier plots were constructed using the datasets from KM-plotter web tool (www.kmplot.com). The Kaplan-Meier curves were compared using a log rank test P. P ≤ 0.05 was considered statistically significant. Supplementary file12 (JPG 1330 KB)

Supplementary Figure 8. Relative expression of (A) miR-122 and (B) BORIS in MDA-MB-231 and MDA-MB-231RR cells compared with non-tumorigenic human breast MCF-10A cells. Bars represent the mean ±1 SD of three independent experiments. *P ≤ 0.05; **P ≤ 0.01; ****P ≤ 0.0001 by Student´s t-test. P ≤ 0.05 was considered statistically significant. Supplementary file13 (JPG 116 KB)

Supplementary Figure 9. High CTCFL expression in TNBC tumors with loss of miR-122 expression is associated to cell invasion and migration. Circular plots of gene ontology (GO) analysis based on (A) molecular function, (B) cellular component, and (C) significantly enrichment KEGG, Reactome, Panther and Wiki pathways from DEGs in CTCFL-overexpressing TNBC tumors (n= 632 genes). P ≤ 0.05 was considered statistically significant. Supplementary file14 (JPG 555 KB)

## Data Availability

The data that support the findings of this study are available on request from the corresponding author.
