# Supplementary material for: Loss of miR-122 promotes cell migration and poor prognosis in triple-negative breast cancer treated by neoadjuvant chemotherapy
Source: Clin Transl Oncol. 2025 Oct 22;28(4):1285–302. doi: 10.1007/s12094-025-04082-x (PMC13009078; doi:10.1007/s12094-025-04082-x)
Supplement: Supplementary file 1 — Supplementary file1 (DOCX 16 KB) [file 12094_2025_4082_MOESM1_ESM.docx]

| **Table S1. Clinical data of TNBC patient population** | | |
| --- | --- | --- |
| **Clinical traits of TNBC patients** | **TNBC cohort** | |
|  | **miR-122**  **upregulated** | **miR-122**  **downregulated** |
|  | **n = 52** | **n = 53** |
| **miR-122 (Mean ± SD)^a^** | -1.26 ± 0.60 | -2.65 ± 0.50 |
| **Age (Mean ± SD)** | 53 ± 10.78 | 58 ± 12.26 |
| **Treatment time (Mean ± SD) ^b^** | 4.75 ± 2.40 | 5.68 ± 14.95 |
| **Vital status** |  |  |
| Alive | 51 (98.1 %) | 47 (88.7 %) |
| Dead | 1 (1.9 %) | 6 (11.3 %) |
| **OS (Mean ± SD)^c^** | 75.91 ± 48.28 | 13.80 ± 7.83 |
| **RFS (Mean ± SD)^c^** | 71.77 ± 47.88 | 13.64 ± 7.49 |
| **Pathologic response^d^** |  |  |
| pCR | 47 (90.4 %) | 50 (94.3 %) |
| pPR/pNR | 5 (9.6 %) | 3 (5.7 %) |
| **ER status^e^** |  |  |
| Positive | 7 (13.5 %) | 7 (13.2 %) |
| Negative | 45 (86.5 %) | 46 (86.8 %) |
| **PR status^f^** |  |  |
| Positive | 7 (13.5 %) | 3 (5.7 %) |
| Negative | 45 (86.5 %) | 50 (94.3 %) |
| **HER2^g^** |  |  |
| Positive | 1 (1.9 %) | 0 (0.0 %) |
| Negative | 51 (98.1 %) | 53 (100 %) |
| **Tumor stage** |  |  |
| Stage I | 10 (19.2 %) | 2 (3.8 %) |
| Stage II | 38 (73.1 %) | 42 (79.2 %) |
| Stage III | 4 (7.7 %) | 9 (17.0 %) |
| Stage IV | 0 (0 %) | 0 (0 %) |
| Stage X | 0 (0 %) | 0 (0 %) |

**^a^** The normalized expression for miR-122, **^b^** Treatment days values in months, **^c^** Overall Survival (OS) and Recurrence-Free Survival (RFS) values in months, **^d^** Pathologic complete response (pCR), pathological partial response (pPR) and pathological non-response (pNR), **^e, f, g^** ER, PR and HER2 status by IHC.
