# Supplementary figures and images for: Loss of miR-122 promotes cell migration and poor prognosis in triple-negative breast cancer treated by neoadjuvant chemotherapy
Source: Clin Transl Oncol. 2025 Oct 22;28(4):1285–302. doi: 10.1007/s12094-025-04082-x (PMC13009078; doi:10.1007/s12094-025-04082-x)

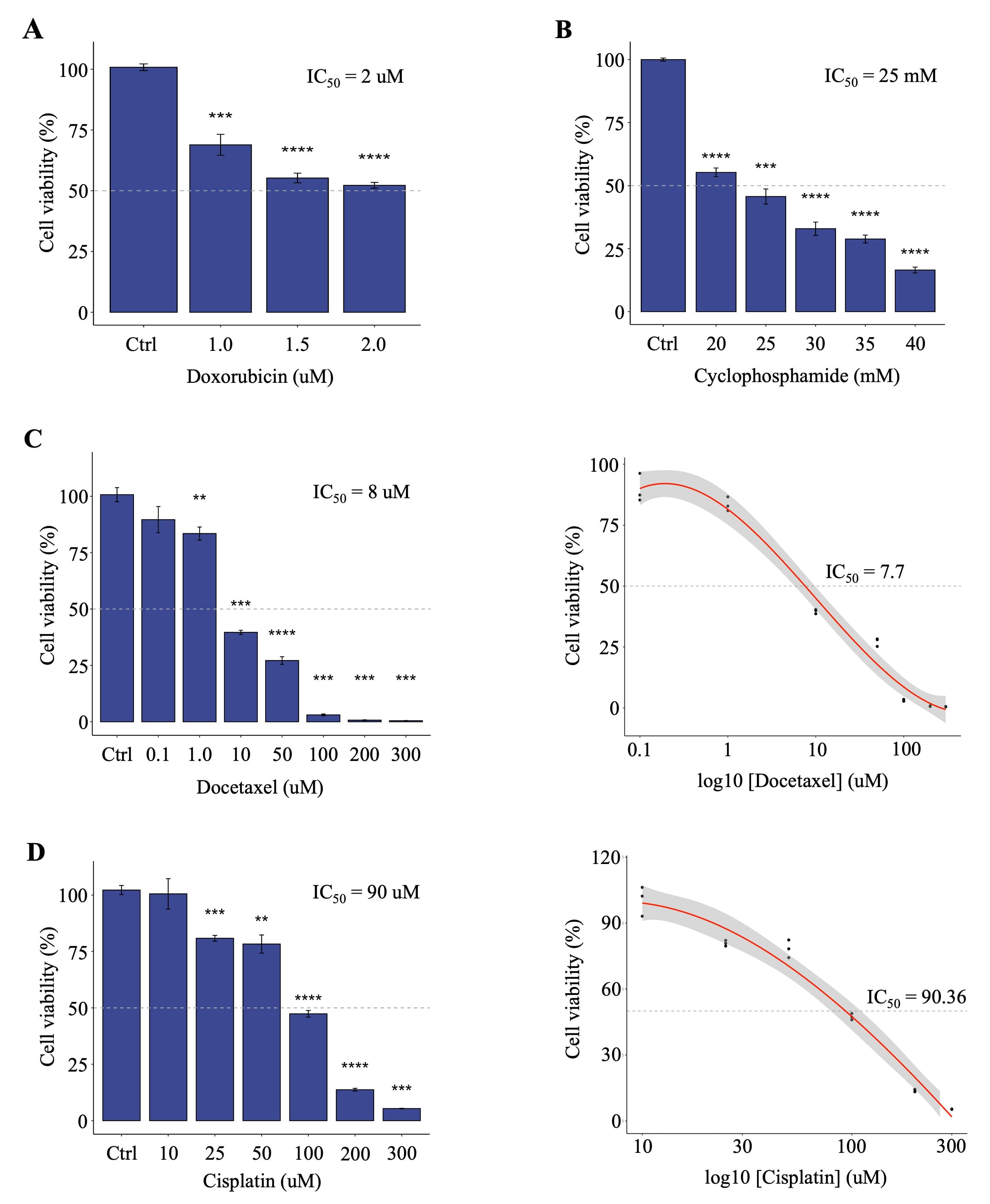

Supplement: Supplementary file 6 — Supplementary Fig. 1. IC50 for drug treatment panel in TNBC cells. Determination of IC50 of (A) doxorubicin, (B) cyclophosphamide, (C) docetaxel and (D) cisplatin in MDA-MB-231 cells treated for 24 hours. The bars represent the average of 3 biological replicates ±1 SD. **P ≤ 0.01, ***P ≤ 0.001, ****P ≤ 0.0001 by Student´s t-test. P ≤ 0.05 was considered statistically significant. Supplementary file6 (JPG 227 KB) [file 12094_2025_4082_MOESM6_ESM.jpg]

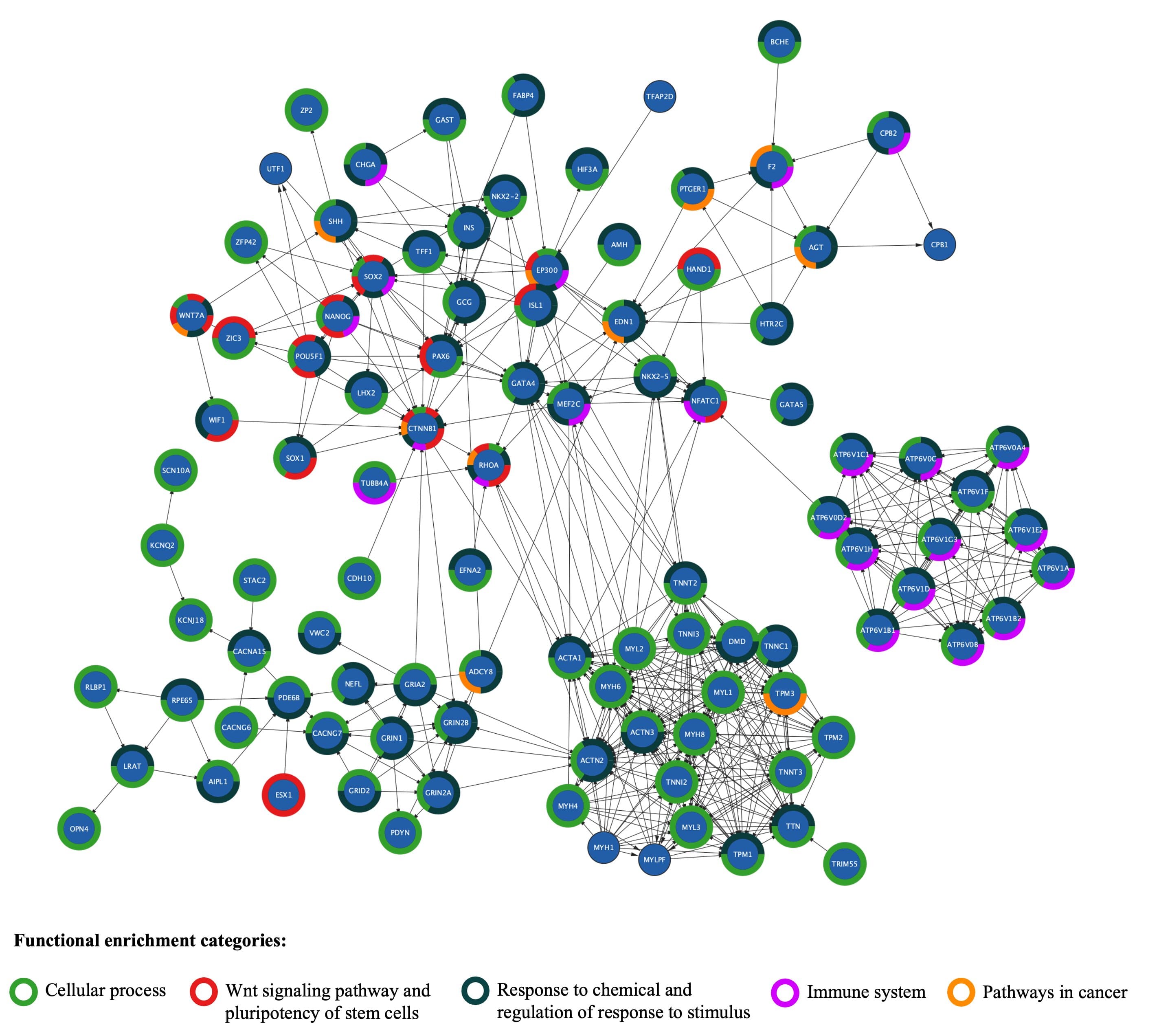

Supplement: Supplementary file 8 — Supplementary Figure 3. Functional enrichment network of blue module. Functional enrichment network analysis from the blue module. The network represents the 25% of 247 genes. Functional enrichment based on Gene Ontology, KEGG pathways, Reactome pathways and WikiPathways categories. Supplementary file8 (JPG 418 KB) [file 12094_2025_4082_MOESM8_ESM.jpg]

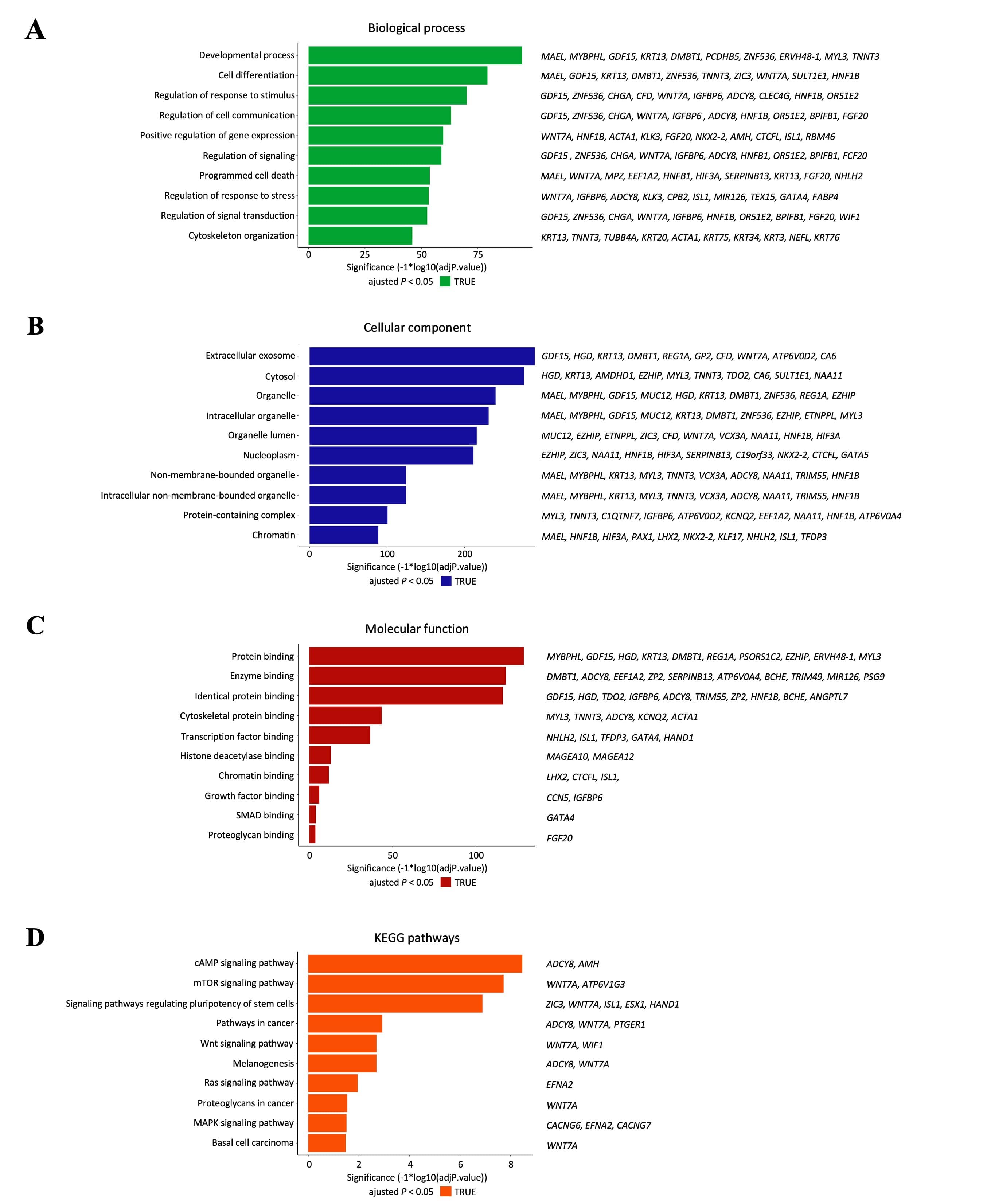

Supplement: Supplementary file 9 — Supplementary Figure 4. Functional enrichment analysis of blue module. Bar plots of gene ontology (GO) analysis from the blue module based on (A) biological process, (B) cellular component, (C) molecular function, and significantly enrichment (D) KEGG pathways from blue module (n= 397 genes). Each bar contains the top 10 genes based on the adjusted P value (to the right of the bar) that enrich the pathway. P ≤ 0.05 was considered statistically significant. Supplementary file9 (JPG 349 KB) [file 12094_2025_4082_MOESM9_ESM.jpg]

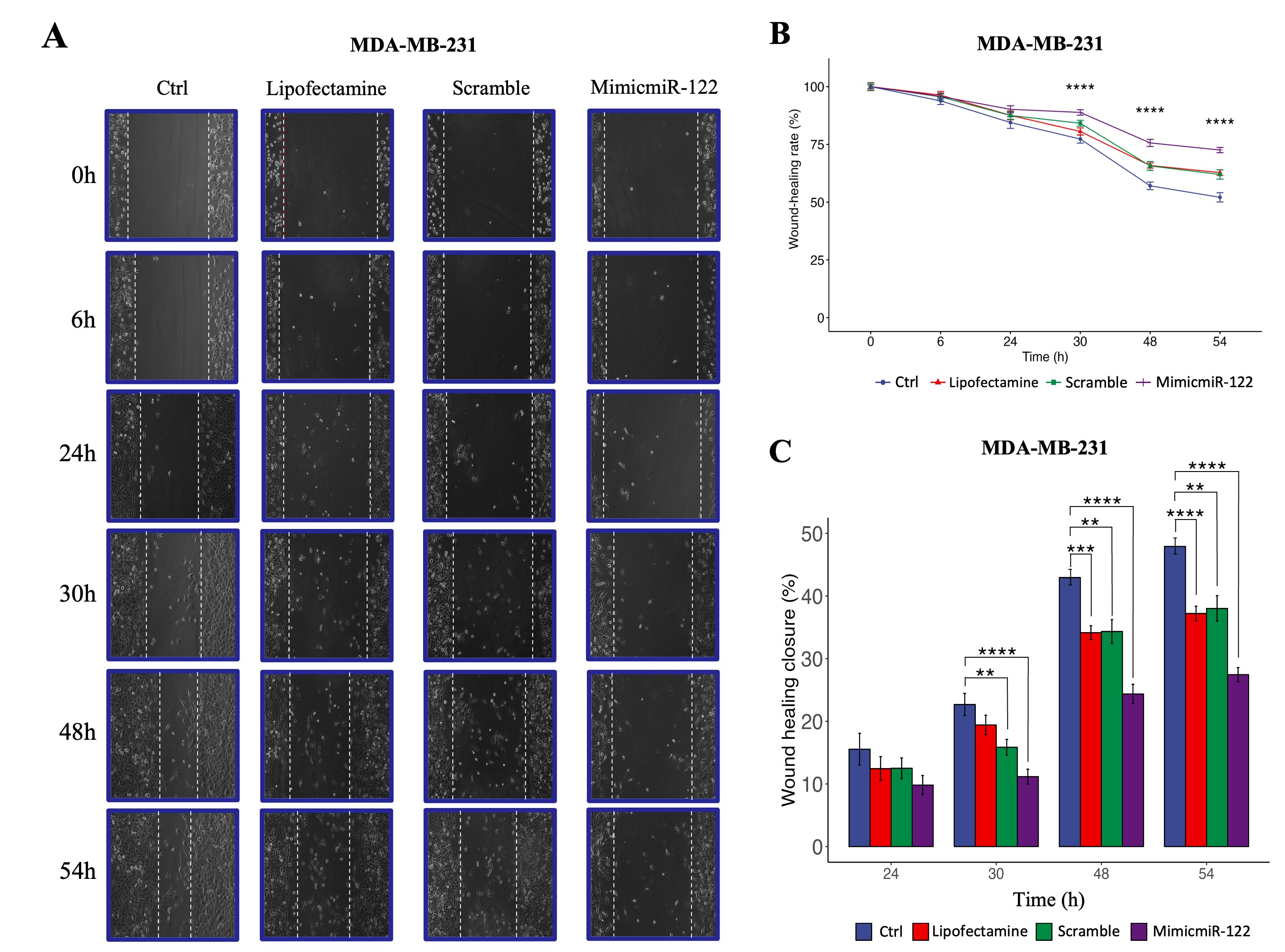

Supplement: Supplementary file 10 — Supplementary Figure 5. Upregulation of miR-122 impairs the migration capabilities of TNBC cells. (A-C) The miR-122 overexpression suppressed wound healing in MDA-MB-231 cells at 30 h, 48 h, and 54 h. The results were compared with control (Ctrl) cells and transfection control cells (Lipofectamine and Scramble). Bars represent the mean ±1 SD of three independent experiments. **P ≤ 0.01; ***P ≤ 0.001; ****P ≤ 0.0001 by Student´s t-test. P ≤ 0.05 was considered statistically significant. Ctrl, non-transfected cells; MimicmiR-122, hsa-miR-122-5p mimics. Supplementary file10 (JPG 651 KB) [file 12094_2025_4082_MOESM10_ESM.jpg]

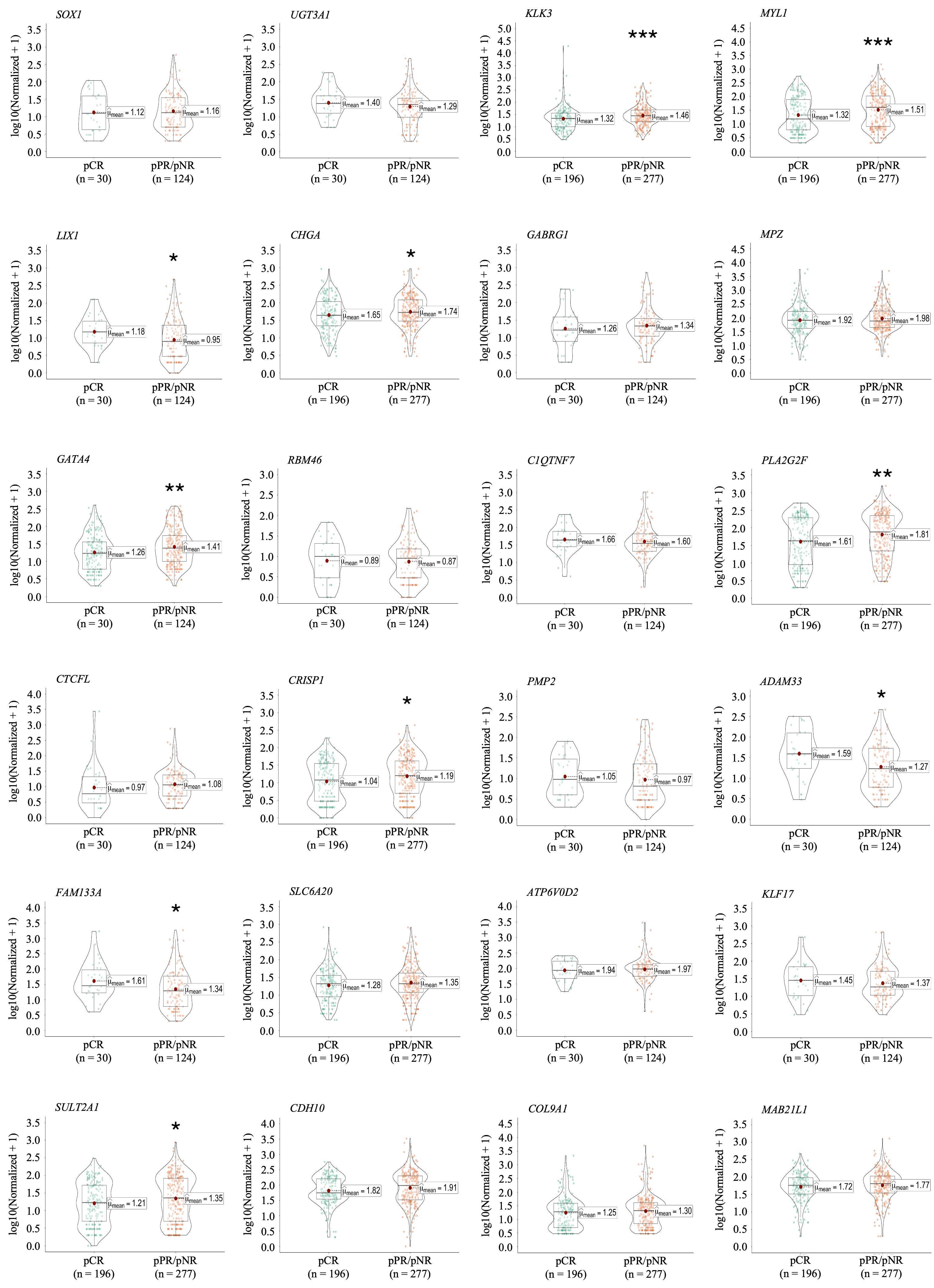

Supplement: Supplementary file 11 — Supplementary Figure 6. The miR-122 target genes were associated to pCR. The expression analysis of the miR-122 target genes in TNBC patients. Red dot in the boxplot indicates the mean of level expression of each gene. *P ≤ 0.05; **P ≤ 0.01 by Wilcoxon test. P ≤ 0.05 was considered statistically significant. pCR, pathological complete response; pPR, pathological partial response; pNR, pathological non-response. Supplementary file11 (JPG 1056 KB) [file 12094_2025_4082_MOESM11_ESM.jpg]

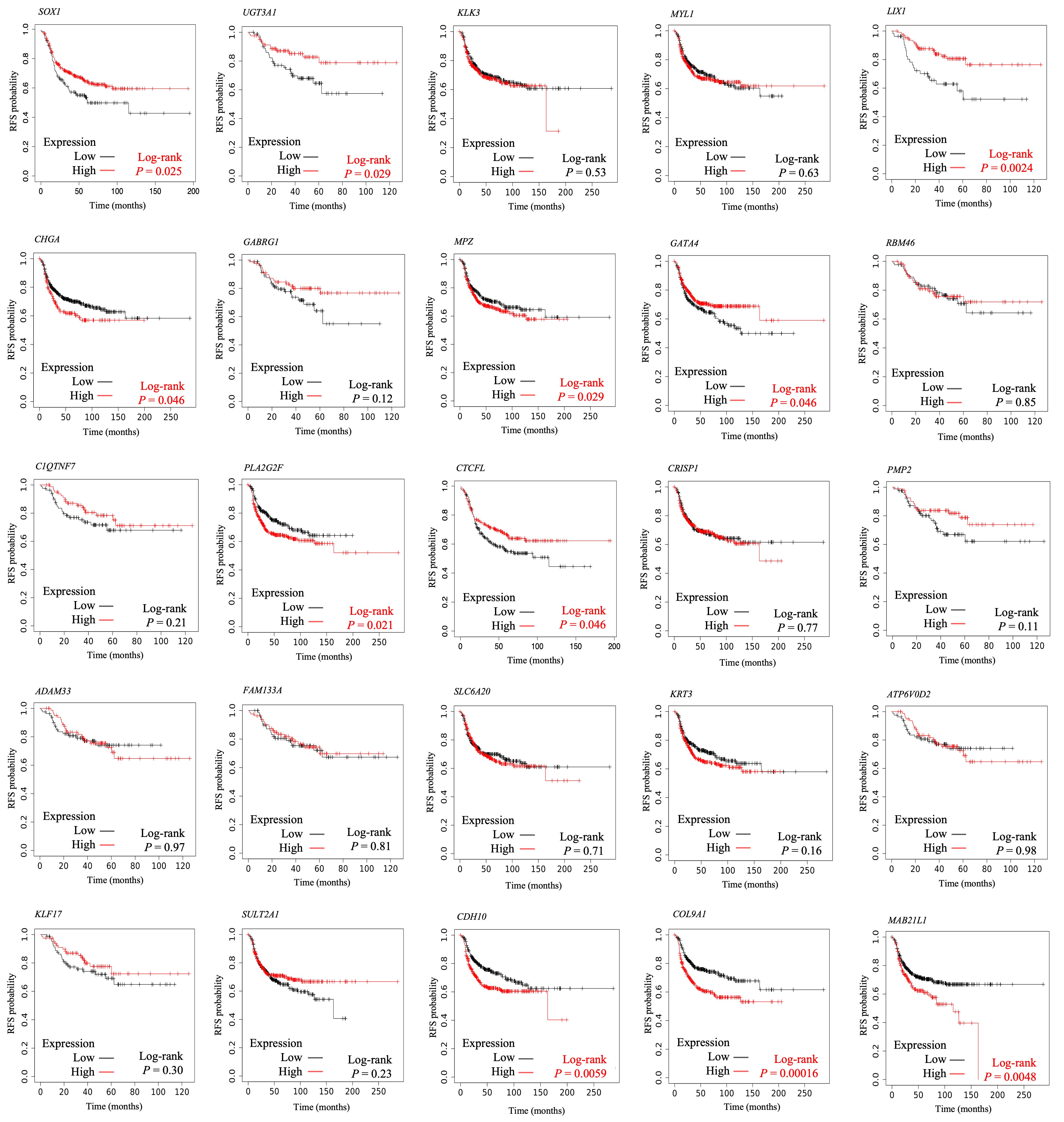

Supplement: Supplementary file 12 — Supplementary Figure 7. The miR-122 target genes predict RFS survival. Kaplan-Meier plots of RFS in TNBC patients to evaluate the predictive potential of the expression of miR-122 target genes. High or low expression levels according to ≥ median or <median of gene expression respectively. The Kaplan-Meier plots were constructed using the datasets from KM-plotter web tool (www.kmplot.com). The Kaplan-Meier curves were compared using a log rank test P. P ≤ 0.05 was considered statistically significant. Supplementary file12 (JPG 1330 KB) [file 12094_2025_4082_MOESM12_ESM.jpg]

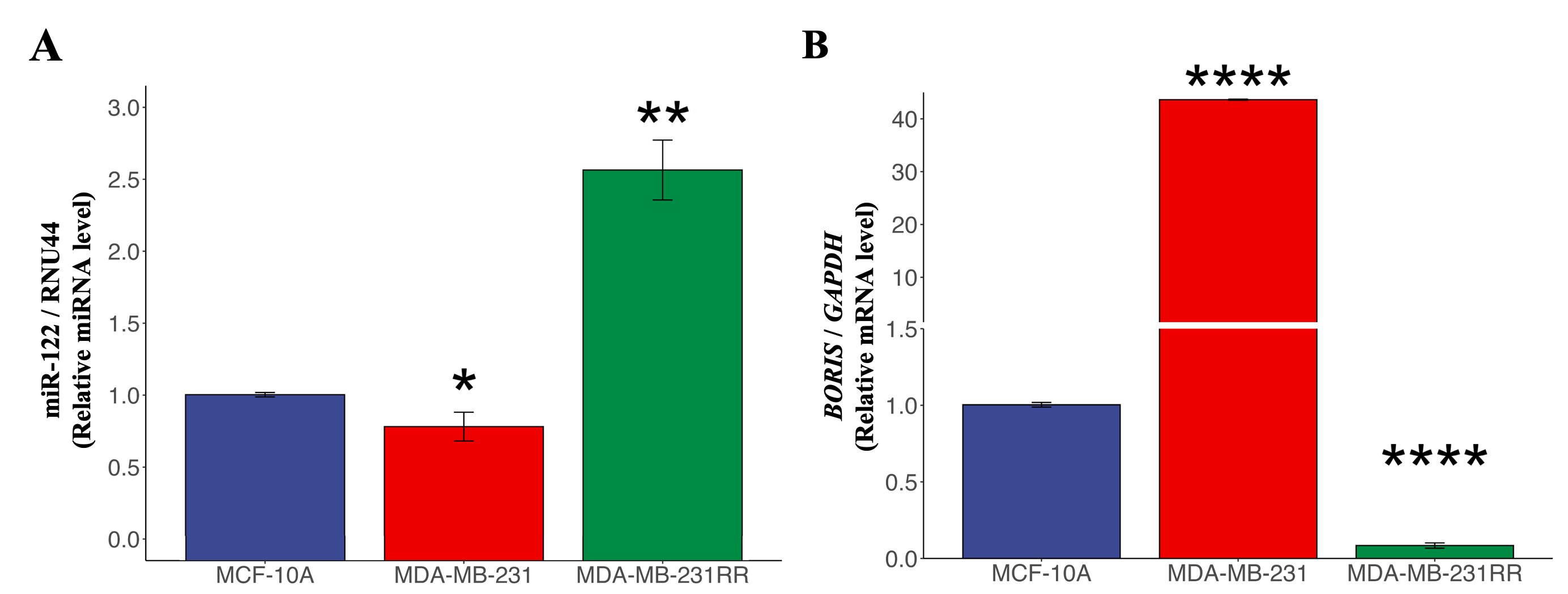

Supplement: Supplementary file 13 — Supplementary Figure 8. Relative expression of (A) miR-122 and (B) BORIS in MDA-MB-231 and MDA-MB-231RR cells compared with non-tumorigenic human breast MCF-10A cells. Bars represent the mean ±1 SD of three independent experiments. *P ≤ 0.05; **P ≤ 0.01; ****P ≤ 0.0001 by Student´s t-test. P ≤ 0.05 was considered statistically significant. Supplementary file13 (JPG 116 KB) [file 12094_2025_4082_MOESM13_ESM.jpg]

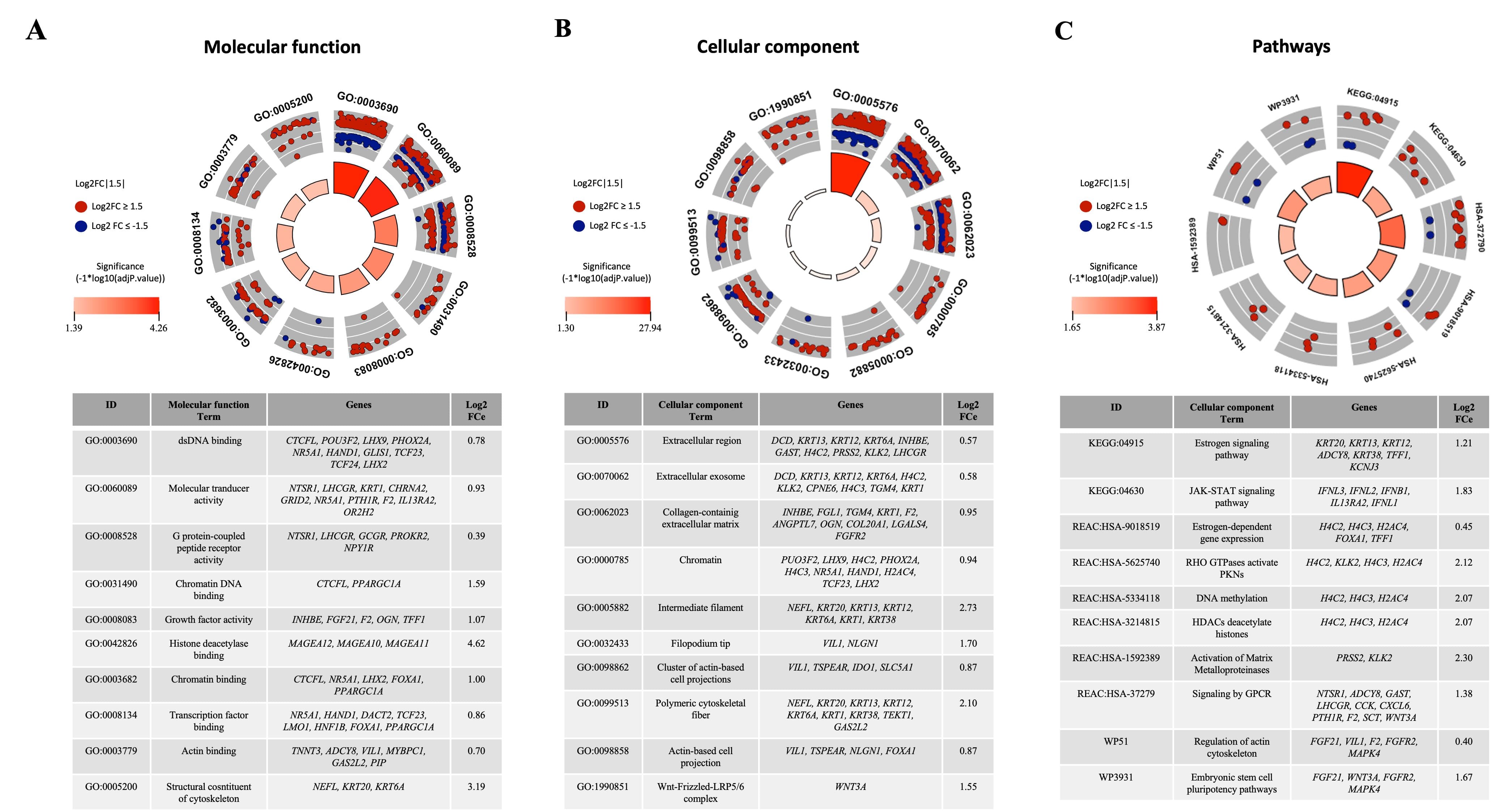

Supplement: Supplementary file 14 — Supplementary Figure 9. High CTCFL expression in TNBC tumors with loss of miR-122 expression is associated to cell invasion and migration. Circular plots of gene ontology (GO) analysis based on (A) molecular function, (B) cellular component, and (C) significantly enrichment KEGG, Reactome, Panther and Wiki pathways from DEGs in CTCFL-overexpressing TNBC tumors (n= 632 genes). P ≤ 0.05 was considered statistically significant. Supplementary file14 (JPG 555 KB) [file 12094_2025_4082_MOESM14_ESM.jpg]
